# Structural analysis of lecithin:cholesterol acyltransferase bound to high density lipoprotein particles

**DOI:** 10.1038/s42003-019-0749-z

**Published:** 2020-01-15

**Authors:** Kelly A. Manthei, Dhabaleswar Patra, Christopher J. Wilson, Maria V. Fawaz, Lolita Piersimoni, Jenny Capua Shenkar, Wenmin Yuan, Philip C. Andrews, John R. Engen, Anna Schwendeman, Melanie D. Ohi, John J. G. Tesmer

**Affiliations:** 1 Life Sciences Institute, University of Michigan, Ann Arbor, MI 48109 USA; 2 Departments of Biological Sciences and of Medicinal Chemistry and Molecular Pharmacology, Purdue University, West Lafayette, IN 47907 USA; 3 Department of Chemistry and Chemical Biology, Northeastern University, Boston, MA 02115 USA; 4 Department of Medicinal Chemistry, University of Michigan, Ann Arbor, MI 48109 USA; 5 Departments of Biological Chemistry, Bioinformatics and Chemistry, University of Michigan, Ann Arbor, MI 48109 USA; 6 Department of Pharmaceutical Science, Biointerfaces Institute, University of Michigan, Ann Arbor, MI 48109 USA; 7 Life Sciences Institute and Department of Cell and Developmental Biology, University of Michigan, Ann Arbor, MI 48109 USA

**Keywords:** Electron microscopy, Biochemistry

## Abstract

Lecithin:cholesterol acyltransferase (LCAT) catalyzes a critical step of reverse cholesterol transport by esterifying cholesterol in high density lipoprotein (HDL) particles. LCAT is activated by apolipoprotein A-I (ApoA-I), which forms a double belt around HDL, however the manner in which LCAT engages its lipidic substrates and ApoA-I in HDL is poorly understood. Here, we used negative stain electron microscopy, crosslinking, and hydrogen-deuterium exchange studies to refine the molecular details of the LCAT–HDL complex. Our data are consistent with LCAT preferentially binding to the edge of discoidal HDL near the boundary between helix 5 and 6 of ApoA-I in a manner that creates a path from the lipid bilayer to the active site of LCAT. Our results provide not only an explanation why LCAT activity diminishes as HDL particles mature, but also direct support for the anti-parallel double belt model of HDL, with LCAT binding preferentially to the helix 4/6 region.

## Abstract (web summary)

Manthei et al. use negative stain electron microscopy, crosslinking, and hydrogen-deuterium exchange to better define the lecithin:cholesterol acyltransferase (LCAT)-high density lipoprotein (HDL) complex. They provide a rationale for why LCAT activity diminishes as HDL particles mature into a spherical shape.

## Introduction

Coronary heart disease is inversely related to high-density lipoprotein (HDL) cholesterol levels and the leading cause of death worldwide. In reverse cholesterol transport, HDLs move cholesterol from atherosclerotic plaques to the liver to be excreted; therefore, a greater understanding and bolstering of this process will guide further treatment and prevention of heart disease. A critical step in reverse cholesterol transport is cholesterol esterification, which is catalyzed by lecithin:cholesterol acyltransferase (LCAT) and promotes cholesterol efflux from macrophages^1^. LCAT binds to HDL and transfers an acyl group from phosphatidylcholine (lecithin) to cholesterol molecules contained within the particle. Cholesteryl ester then partitions to the interior of HDLs, thus promoting further cholesterol efflux from arterial plaques and driving HDL maturation from discoidal pre-β HDL to spherical α-HDL^2,3^. LCAT comprises α/β-hydrolase, membrane-binding, and cap domains, the latter of which contains an active site lid that controls access to the active site^4–7^. The extreme N terminus of LCAT is thus far not ordered in crystal structures, but, along with the membrane-binding domain (MBD), constitute important membrane-binding determinants^5,8,9^. Over 90 genetic mutations in LCAT have been described that lead to one of two characterized diseases: fish eye disease and familial LCAT deficiency^10,11^. Both are characterized by low levels of HDL cholesterol and corneal opacities; however, familial LCAT deficiency presents additional serious symptoms including anemia, proteinuria, and ultimately renal failure^12,13^. Interestingly, familial LCAT deficiency patients do not have an increased risk for cardiovascular disease, and furthermore experiments in mice failed to show an increase in reverse cholesterol transport upon overexpression of human LCAT, even with an increase in HDL cholesterol^12,14^. This, coupled with data from clinical treatments designed to raise HDL cholesterol which failed to protect against heart disease, has led to questions about our understanding of reverse cholesterol transport and the importance of LCAT in the process^15^.

LCAT is activated by ApoA-I, the most abundant apolipoprotein in HDL^16,17^. ApoA-I contains an N-terminal globular domain followed by ten tandem amphipathic α-helices, and current models indicate that two monomers wrap anti-parallel around a lipid bilayer in discoidal HDL, forming a double belt^18^. The double belt is centered on helix 5 (5/5 registry, wherein helix 5 from one chain interacts with helix 5 of the other) which maximizes intermolecular salt bridges; however, other registries have also been reported at low abundance, such as a 5/2 and a 5/4 model^18–22^. There is extensive evidence that the central helices (4–7) of ApoA-I are responsible for LCAT binding and activation, in particular helix 6, which spans residues 143–164 (ref. ^17^). For example, three conserved arginines (residues 149, 153, and 160 in helix 6) are reported to be critical for LCAT activation^23^. Further, mutations in ApoA-I that are deficient in LCAT activation cluster to helices 6 and 7 and to helix 4 which aligns with helix 6 in the 5/5 double belt model^19,24^. Previous hydrogen–deuterium exchange mass spectrometry (HDX-MS) experiments have shown that a region within helices 6 and 7 is protected from deuterium exchange in the presence of LCAT^25^. Recently, specific crosslinks between LCAT and ApoA-I were reported, and two of the ApoA-I crosslinks were at Lys118 (helix 4–5 junction) and Lys140 (helix 5–6 junction), which are within 10 Å of each other on distinct ApoA-I chains in the double belt model^19^. A third crosslink was located in helix 7.

We previously identified hydrophobic residues in LCAT associated with fish eye disease that form a latch for the active site lid^5^. Because fish eye disease mutations are known to specifically affect activity on HDLs, we hypothesized that lid displacement is an important feature of ApoA-I activation. Lid latch mutants were defective in acyltransferase activity, yet revealed no change in HDL binding, which is consistent with the idea that LCAT initially binds to the lipid bilayer via its N-terminal hydrophobic anchor which is connected to the catalytic core of the enzyme through a disordered linker. Indeed, LCAT binds to different HDL subspecies and HDLs with ApoA-II with similar affinity, and changes to the central helices of ApoA-I appear to affect reactivity but not affinity^19,23,26,27^.

Direct visualization of LCAT bound to HDL complexes is needed in order to gain new insights into how a phospholipase engages its physiological target and genetic disease influences the interface. However, both LCAT and HDLs are highly dynamic entities, hampering high-resolution crystallographic analysis. Thus we turned to negative stain electron microscopy (EM), which we validated with HDX-MS and crosslinking coupled with mass spectrometry (XL-MS). Our results show that LCAT preferentially interacts with the edge of HDL particles in a manner consistent with making direct interactions with helix 4/6 region of the ApoA-I double belt where LCAT gains access to the acyl tails of lipids at the edge of the protein-delimited lipid bilayer.

## Results

### Negative stain electron microscopy of the LCAT–HDL complex

**Fig. 1:**
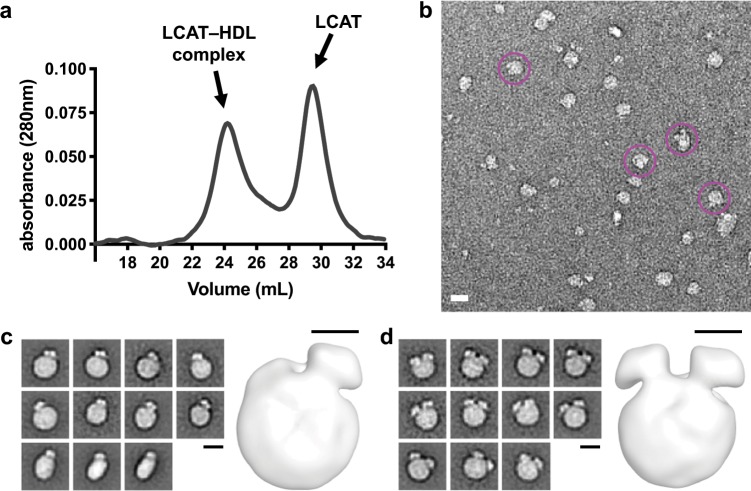
Negative stain EM visualizes one or two LCAT molecules preferentially bound to HDL. **a** The LCAT–HDL complex is separated from excess free LCAT and HDL via SEC. The peak of the complex was then imaged. The shoulder after the complex peak corresponds to free HDL. **b** A raw micrograph of negative stain EM showing individual particles of the LCAT–HDL complex. Pink circles show particles with 2:1 LCAT:HDL. The scale bar is 20 nm. **c**, **d** Selected class averages (left) and a 3D reconstruction (right) of particles revealing 1:1 (**c**) and 2:1 (**d**) LCAT:HDL stoichiometry. No side views are shown for the 2:1 complex as they would be similar to side views for a 1:1 complex. The black scale bar for the 2D classes is 10 nm and for the 3D models it equals 5 nm. See Supplementary Fig. 1 for all class averages.

Recombinant HDL particles composed of a 100:1 molar ratio of lipid:human ApoA-I were incubated with an ~10-fold molar excess of recombinant human LCAT. This large molar excess is not physiological, but facilitated isolation of complexes by size exclusion chromatography (SEC) (Fig. 1a). The peak fraction was then imaged by negative stain EM (Fig. 1b). We generated reference-free negative stain two-dimensional (2D) class averages revealing three dominant populations: HDL alone (~15%), one LCAT per HDL (~40%), and two LCATs per HDL (~45%) (Fig. 1c, d, Supplementary Fig. 1a) although there is evidence for a small proportion of complexes (~1%) having three or more LCAT particles bound in the dataset (Supplementary Fig. 1b). These percentages are likely underestimated for the 1:1 and 2:1 LCAT:HDL classes, because bound LCAT will not always be visible in side views of the complex. However, the majority of the views obtained are in a similar orientation with lipid head groups of the disc preferentially adhering to the carbon support (Supplementary Fig. 1c). An ab initio model was generated with VIPER^28^ and then used as an initial reference for three-dimensional (3D) classification in RELION^29^. Two stable classes corresponding to the 1:1 and 2:1 LCAT:HDL models were generated from the 3D classification and then refined in RELION to produce the final maps (Fig. 1c, d)^29^.

In the 2D and 3D models, LCAT binds exclusively to the side of the discoidal HDLs, which is consistent with its predicted interactions with ApoA-I and also with it gaining access to cholesterol and the acyl chains of the lipids at the edge the bilayer. Furthermore, the staggered orientation (~60° from each other in the 3D model) of the two LCATs in the 2:1 complex is consistent with LCAT preferentially binding to the helix 4/6 region of ApoA-I in the 5/5 double belt model of HDL, wherein helix 6 from each ApoA-I monomer are also spaced ~60° apart. In the majority of 2D classes, the angular spacing between the two LCATs ranges from 49° to 87°, with an average of 63 ± 10° (Supplementary Fig. 2a). In some classes, such as 44 and 72, the spacing appears even wider, up to 127° (Supplementary Fig. 1). In addition to being offset by an average of 60° from each other around the perimeter of the HDL disc, the 3D reconstructions indicate that when two LCAT molecules bind to the side of the disc they are offset from each other by ~15 Å along the central axis of the disc, consistent with them being bound preferentially to distinct but twofold related ApoA-I chains spaced ~12 Å apart in the double belt model (Supplementary Fig. 2b). In many of the 2D classes there appear to be two distinct lobes within the concave density for LCAT. In some classes it also appears that the LCAT is connected to the HDL towards what would be the helix 5–6 junction in the 5/5 double belt model.

The variation in the overall angular separation between LCAT molecules could be explained in a number of different ways, with one hypothesis being that LCAT varies its binding between the helix 5 and 6 junction or the helix 6 and 7 junction (Supplementary Fig. 2a). Extra density between the two bound LCATs in the 3D model could also be due to a looped ApoA-I belt^30^ that consists of helix 5 and part of 6, and such looping out could also change the angle between LCATs bound to helix 6. Another option is due to negative stain artifact, as the complex is flattened in the staining and imaging process. An additional possibility is that the angular change is due to independent rotation of each ApoA-I monomer to adopt different helical registries (Supplementary Fig. 2c)^19–21,31^. The proposed 5/2 helical registry^19,20^ is expected to be at low abundance and would put the two helix 6 locations on opposite sides of the HDL whereas the 5/4 registry^22^ would have a wider separation than the 5/5 orientation (Supplementary Fig. 2c). Thus, these registries cannot account for most of the observed classes of particles, although they could explain classes with wider angular separation.

### XL-MS defines proximal lysine residues within the LCAT–HDL complex

**Fig. 2:**
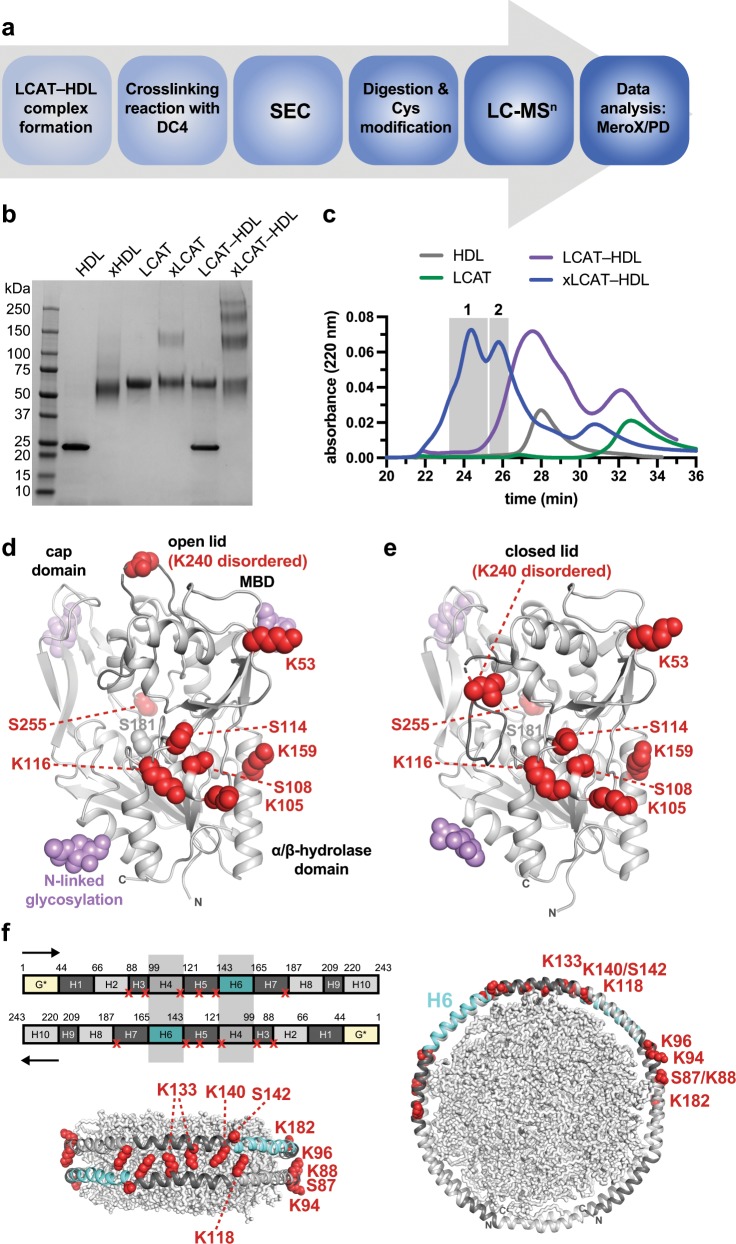
LCAT–HDL crosslinking implicates preferential LCAT–HDL interactions. **a** XL-MS workflow, see Methods for further details. PD refers to the XLinkX node of Proteome Discoverer. **b** SDS-PAGE gel of LCAT–HDL crosslinking experiments. Each lane is described above, with the protein(s) in that experiment, and an x specifying an experiment that included DC4 crosslinker. The xLCAT–HDL lane reveals new higher molecular weight species corresponding to crosslinked products. **c** SEC results for the crosslinked complex, compared to LCAT or HDL alone. The two shaded peaks were then subjected to MS and peptide identification, with results in Table 1. **d**, **e** Crosslinked LCAT residues are shown in red spheres on the **d** open (PDB code 6MVD) and **e** closed (PDB code 5TXF) LCAT crystal structures. Lys240 is shown with a nearby residue as it is disordered in all crystal structures. The dynamic LCAT lid is a darker gray to highlight the movement between the two structures and the Ser181 side chain in gray spheres as a marker for the active site. N-linked NAG sugars are shown with purple spheres and are expected to be excluded from the LCAT–HDL interface. **f** ApoA-I crosslinks are mapped on the ApoA-I primary sequence with two proteins depicting the double belt orientation as shown below and to the right^18^. Crosslinks are shown with a red x on the sequence and red spheres in the structures. ApoA-I helices (H1–10) are colored the same in both subunits. The yellow N-terminal region of ApoA-I is not included in the double belt model. The shaded boxes indicate the hypothesized LCAT-binding location. Lipids are shown as gray sticks in the middle of the ApoA-I belt in each structure.

To further refine the orientation of LCAT within the maps we used XL-MS to determine residues in close proximity within our LCAT–HDL preparation. HDLs were incubated with an excess of LCAT then crosslinked with DC4, an amine-selective mass spectrometry (MS)-cleavable crosslinker with a maximum spacer arm length of 18 Å^32^ (Fig. 2). Crosslinking with DC4 revealed higher-mobility bands on an SDS-PAGE gel that were subsequently separated via SEC and analyzed via MS (Fig. 2b, c, Supplementary Fig. 3a). Two separate peaks consistently eluted that corresponded to crosslinked complex, which were analyzed individually with both Proteome Discoverer (PD) and MeroX to produce the full dataset (Table 1, Supplementary Table 1, Supplementary Figs. 3b–7). No major differences were observed in the results from the two analyzed peaks. We observed 15 unique crosslinks between LCAT and ApoA-I, with the majority of the LCAT crosslinks occurring on a common surface of LCAT spanning the MBD, the lid, the αA–αA′ loop, and the α/β-hydrolase domain (Fig. 2d, e). This surface also includes many hydrophobic residues proposed to interact with HDLs^5,33^ as well as residues reported to crosslink in another study using bissulfosuccinimidyl suberate (BS3, spacer arm of 11.4 Å)^19^. For ApoA-I, crosslinks with LCAT were all found within the known LCAT-binding hotspot spanning helices 3–7 (refs. ^17,19^), consistent with the range of angles observed in the 2D class averages (Fig. 2f, Supplementary Fig. 2a). Crosslinks are conspicuously absent in the helix 4/6 segments of ApoA-I even though there are two consecutive lysine residues in helix 4. It is anticipated that LCAT preferentially binds to this region and protects these residues from the DC4 crosslinker. We also observed intra-LCAT and intra-ApoA-I crosslinks, as well as crosslinks between peptides containing the same reactive lysine signifying inter-LCAT and inter-ApoA-I crosslinks (Supplementary Table 1). The inter-LCAT crosslink is between Lys105/Ser108 in the 2:1 complex (Table 1, Supplementary Fig. 7), suggesting that at least some of the LCAT–HDL complexes have the corresponding surfaces of LCAT facing each other in the complex, consistent with twofold symmetry.

**Table 1:** XL-MS of the LCAT–HDL complex using DC4.

Crosslink		Crosslinked peptide sequence^a^		Peak 1^b^					Peak 2				
LCAT	ApoA-I	LCAT	ApoA-I	Score^c^		CSM^d^		Rep	Score		CSM		Rep
K240	K140/S142	239-L**K**EEQR-244	137-LQE**K**L**S**PLGEEMR-149	263	148	27	8	3	213	133	6	2	2
K105/S108	K140/S142	100-VPGFG**K**TY**S**VE-110	137-LQE**K**L**S**PLGEEMR-149	203	91	16	8	3	206	35	1	2	1
K105/S108	K118	100-VPGFG**K**TY**S**VE-110	117-Q**K**VEPLRAE-125	183	142	10	3	2	153		1		1
K240	K182	239-L**K**EEQR-244	178-LEAL**K**ENGGAR-188	158		6		2	191		2		2
K105/S108	K94/K96	100-VPGFG**K**TY**S**VE-110	92-EV**K**A**K**VQPYLDDFQK-106	152		4		3	144	56	2	3	2
K105/S108	K133	100-VPGFG**K**TY**S**VE-110	132-Q**K**LHE-136	107	64	2	2	1	101	51	2	1	2
K159	K182	159-**K**LAGLVEE-166	178-LEAL**K**ENGGAR-188	105	53	3	2	2	55	57	2	2	2
K159	K140/S142	159-**K**LAGLVEE-166	137-LQE**K**L**S**PLGEEMR-149	92	172	16	13	3	42	139	2	1	1
K159	K133	159-**K**LAGLVEE-166	132-Q**K**LHE-136	37	107	5	1	3	38		2		2
K159	K118	159-**K**LAGLVEE-166	117-Q**K**VEPLRAELQEGAR-131	32	107	6	6	3					
K53	K140/S142	53-**K**TEDFFTI-60	137-LQE**K**L**S**PLGEEMR-149		143		14	1^e^		145		3	1
K53	K118	53-**K**TEDFFTI-60	117-Q**K**VEPLR-123		124		4	2					
S114/K116	S87/K88	111-YLD**S**S**K**LAGY-120	86-m**SK**DLEEVKA-95		115		23	2		111		6	2
S255	K140	252-MFP**S**R-256	137-LQE**K**LSPLGEEMR-149		64		109	2		64		42	2
S255	K118	252-MFP**S**R-256	117-Q**K**VEPLR-123		62		7	2		40		1	1
**LCAT**	**LCAT**	**LCAT**	**LCAT**	**Score**		**CSM**		**Rep**	**Score**		**CSM**		**Rep**
K105/S108^f^	K105/S108	97-QIRVPGFG**K**TY**S**VE-110	100-VPGFG**K**TY**S**VE-110		139		14	3		145		8	3

^a^Peptide sequences with the highest score

^b^Each peak refers to the highlighted portion of the crosslinked SEC chromatogram in Fig. 2b

^c^The score and spectra in the left column are from Proteome Discoverer, and the right column from MeroX

^d^Crosslinked peptide-spectra matches

^e^LCAT-K53 ApoA-I-K140 crosslink was found in only one replicate for each peak, but in different replicates between the two peaks

^f^Crosslinks between identical residues must be between two LCATs

m: oxidized methionine; Rep: replicates

The XL-MS data also reflect the dynamic nature of the LCAT–HDL complex observed in the 2D class averages (Supplementary Fig. 1a, b). There are multiple hyperconnected lysine residues, which are diagnostic for flexible domains. An example is LCAT-Lys240, a disordered residue in the dynamic lid region, which crosslinks to ApoA-I-Lys140 and Lys182 using DC4 (this study), and ApoA-I-Lys140, Lys182, and Lys118 using BS3 (ref. ^19^). Conversely, LCAT-Lys159 is located in a stable helix of the α/β-hydrolase domain and reacts with ApoA-I-Lys118, Lys133, Lys140, and Lys182, suggesting that these regions of ApoA-I are highly dynamic. It is not possible to come up with a single docking model in which LCAT-Lys240 and Lys159 can get within range of either hotspot on ApoA-I, implying heterogeneity in LCAT binding. However, the fact that most of the crosslinks are found in discrete regions of the ApoA-I belt on either side of the helix 4/6 double belt segment indicates a preferred binding site for LCAT on HDL.

### Change in dynamics upon LCAT binding to HDL via HDX-MS

Although HDX-MS has previously been reported for ApoA-I in HDL^34–37^, we obtained HDX-MS data for both LCAT and ApoA-I, both before and after LCAT–HDL complex formation. The deuteration of each monitored peptide was measured after 10 s, 30 s, 3 min, 10 min, and 30 min of exposure to deuterated buffer (Supplementary Figs. 8 and 9). For LCAT there was poor MS coverage for the N terminus, whereas for ApoA-I the middle of the protein was poorly covered. HDX data comparing free and bound LCAT and ApoA-I are summarized in individual peptide deuterium uptake curves (Supplementary Fig. 10). For free ApoA-I, there were high levels of exchange across most of the protein, especially at both termini and the first two helices, in agreement with previously published data^34–37^ (Supplementary Fig. 11). Unfortunately, this high rate of exchange made it difficult to observe protection attributable to the presence of LCAT, especially at later time points (Supplementary Fig. 11). Furthermore, the MS coverage for ApoA-I between residues 115–200 was poor, and the peptides that were observed did not change in the presence of LCAT. We did observe protection in the first two ApoA-I helices (residues 39–70) at shorter time points, which we interpret to be a result of LCAT stabilizing the HDLs overall.

**Fig. 3:**
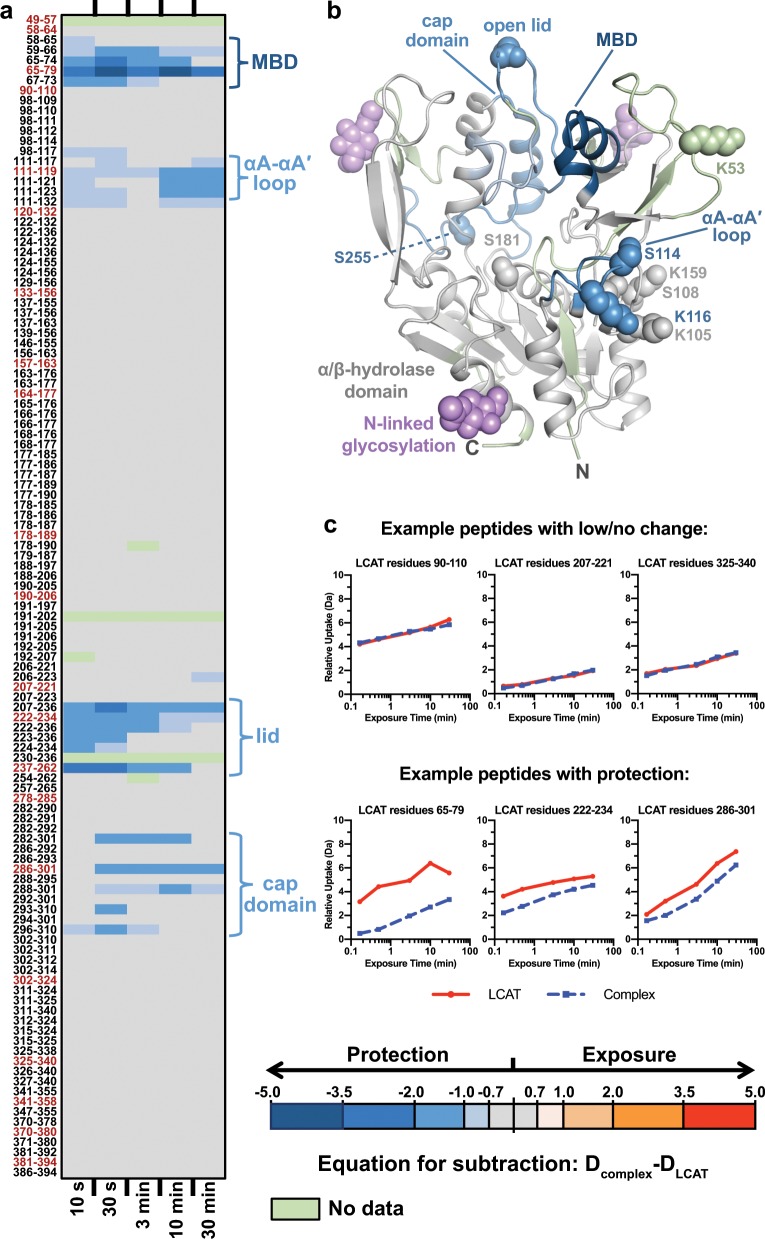
HDX-MS reveals HDL-induced protection on LCAT. **a** The panel shows the differences in relative deuterium uptake between LCAT alone and LCAT bound to HDL for all peptides identified and followed by HDX-MS. The exchange time is indicated at the bottom of the panel, increasing from 10 s to 30 min from left to right. All differences are shown in Da and are color-coded according to the scale at the bottom right. Down the left side are the residue numbers of each peptide fragment, arranged from N to C terminus (top to bottom). Peptides highlighted in red are representative of the differences in relative deuterium incorporation and were plotted onto **b** an open X-ray crystal structure of LCAT (PDB entry 6MVD) at the 10 min timepoint using PyMOL. **c** Representative deuterium uptake curves for LCAT peptides that show examples of low/no change in deuterium incorporation, and decreased exchange due to protection from HDL binding. All of the deuterium incorporation curves used to create this figure are also shown in Supplementary Fig. 10.

For LCAT alone, we saw a pattern similar to that previously reported^5^ with the MBD (specifically residues 59–73), αA-αA′ loop (residues 111–123), lid region (residues 222–262), and the back of the cap domain near the lid (residues 286–301) having the highest exchange rates (Supplementary Fig. 12). These regions exchanged less while in complex with HDL (Fig. 3), suggesting that they either directly interact with HDLs or that they become stabilized because the active site lid is retracted and held in a more stable conformation that allows lipid substrates to access the active site, or both. Because we observe crosslinks within the αA–αA′ loop (Ser114/Lys116), lid (Lys240), and adjacent MBD peptides (Lys53), and these sites are all on the same face of LCAT, we interpret these regions to be stabilized by direct interactions with HDL. Overall, the regions stabilized as measured by HDX were similar to those of LCAT when reacted with a fluorophosphonate suicide inhibitor (IDFP)^5^, implying that HDL-bound LCAT could adopt a conformation similar to that of fluorophosphonate-bound LCAT determined by X-ray crystallography. This therefore suggests that this crystallographic model would be appropriate to place in the negative stain envelope.

## Discussion

To better define how LCAT interacts with HDL, we analyzed their complex with negative stain EM, XL-MS, and HDX-MS. Our negative stain EM data show that LCAT binds at the ApoA-I delimited boundary of the HDL disc, in agreement with ApoA-I being a direct activator of LCAT. When two molecules of LCAT are bound, they are separated by ~60° around the perimeter of the HDL consistent with LCAT binding close to the helix 5–6 junction (or the 4–5 junction) in an ApoA-I double belt model. The XL-MS results indicate that there are hotspots on ApoA-I on either side of the helix 4/6 region that are likewise consistent with the helix 4/6 segment being the preferred binding site for LCAT. Previous biochemical studies and known ApoA-I mutations also implicate helices 3–7 and the 4/6 region in binding LCAT^17,19,23–25^. When LCAT is bound to HDL, changes in dynamics measured by HDX-MS mirror those of LCAT when bound to IDFP, which appears to induce the enzyme to adopt an open lid structure that dramatically stabilizes regions surrounding the active site^5,6^. The LCAT·IDFP crystal structure is thus a reasonable atomic model for LCAT, at least at low resolution, that we can dock to a double belt model of HDL with a 5/5 ApoA-I registry.

To build a low-resolution model of the LCAT–HDL complex, we first considered prior structural and biochemical knowledge. LCAT has four sites at which Asn-linked glycosylation occurs, and the binding site of an agonistic Fab antibody is found at the interface of the α/β-hydrolase and cap domains of LCAT^4^. These surfaces of LCAT therefore cannot interact directly with or sterically occlude HDL. The MBD and extreme N terminus of LCAT are well-established membrane-binding determinants and need to be positioned in a way where they can engage the lipid core of the HDL (the first 20 amino acids of LCAT are however disordered in crystal structures). Finally, the active site of the enzyme needs to be positioned in a manner such that it has access to lipid substrates in the HDL while excluding water, which would otherwise lead to a non-productive phospholipase reaction.

The 3D EM density for LCAT has an ellipsoid shape, into which we centered the model of the LCAT activated structure (PDB entry 6MVD chain A)^6^. If one faces the active site of the enzyme towards the HDL edge, there is a concave cavity consistent with the EM map. When positioned this way, the lysine hotspot of LCAT consisting of Lys53, 105, 116, and 159 cannot react with all the regions on ApoA-I indicated by crosslinks due to distance constraints. However, they can easily be placed within 30 Å with lysine hotspots at the helix 4–5 and helix 5–6 boundaries of ApoA-I consisting of Lys118, 133, and 140 (Supplementary Table 2). This interface is also in agreement with the HDX-MS data, where stabilized regions of the αA–αA′ loop, lid, and MBD are all able to contact HDL. The MBD in this orientation is poised to interact with the lipid monolayer adjacent to helix 6, and the first ordered N-terminal residue in the LCAT crystal structure projects such that the extreme N-terminal membrane-binding anchor can easily reach the lipid monolayer on the opposite side of the HDL, near the helix 4–5 junction. LCAT-Lys105 makes a reasonably close approach to its symmetry-related position in the LCAT positioned in the other lobe of EM density that would allow for the observed Lys105-Lys105 inter-LCAT crosslink (Table 1). If the LCAT were rotated 180° so that the MBD and N terminus interacted with the opposite sides of the HDL monolayers, the hotspot at the helix 3/7 region of ApoA-1 (Lys88, 94, 96, 182) remains too distant from most of the reactive residues in LCAT, unless one postulates a register shift in the ApoA-I double belt. Furthermore, superposition shows that the agonistic Fab fragment^4^ would sterically occlude LCAT binding to HDL in this configuration.

**Fig. 4:**
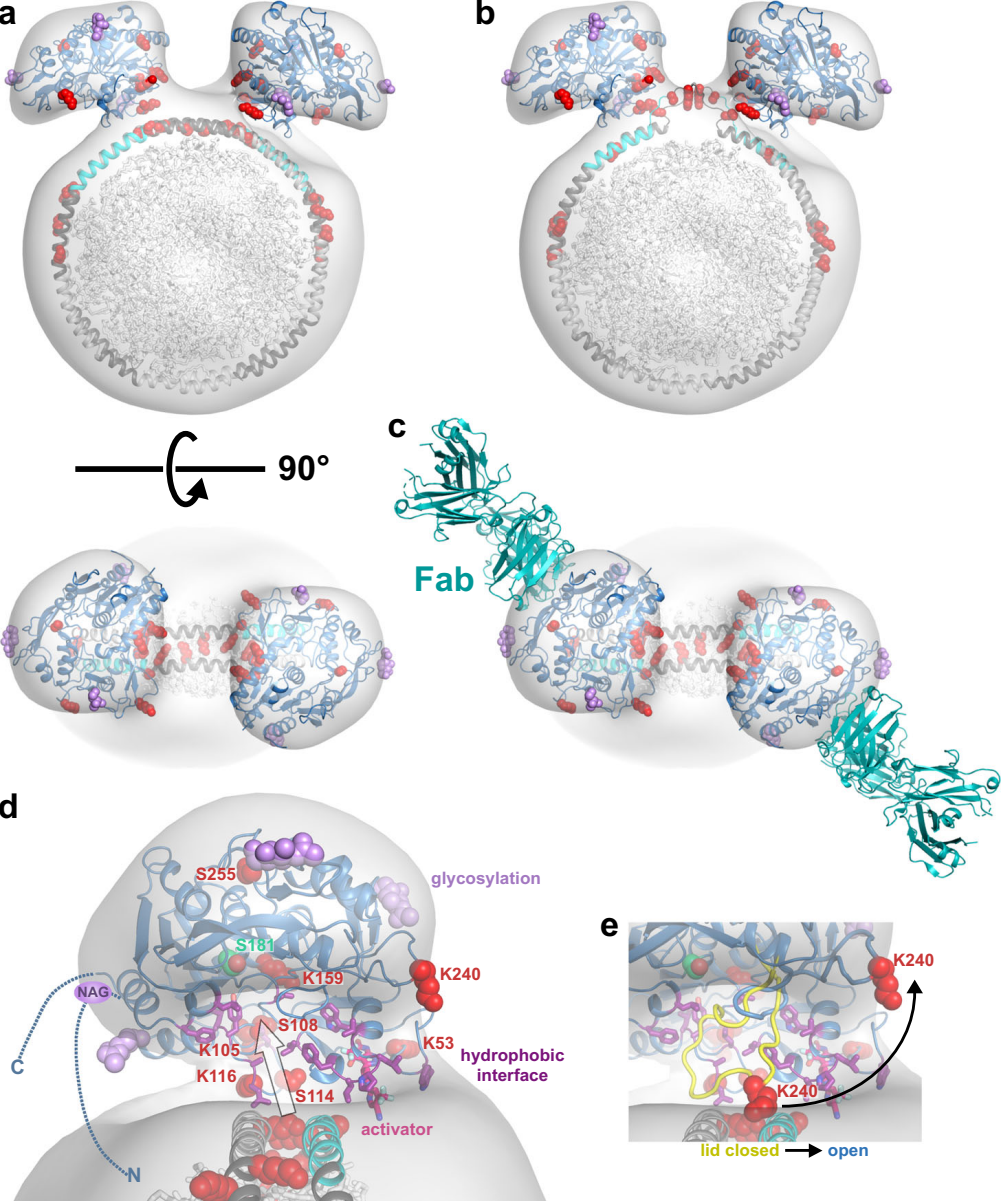
A model for how LCAT binds to HDL. **a**, **b** Activated LCAT (PDB code 6MVD) was docked into each lobe of the 2:1 3D reconstruction with C2 symmetry, with the double belt^18^ (**a**) and looped belt^30^ (**b**) model for HDL placed in the HDL density. **c** The model is compatible with the agonist Fab antibody binding to LCAT^4^. **d** A close-up of the concave surface that allows lipidic substrate to move in (path shown with arrow) and out of the active site (Ser181 in green) from HDL. The hydrophobic surface expected to bind to HDL is depicted with residues in purple sticks. The N and C terminus are disordered and depicted as dashed lines, which includes a glycosylation site (NAG) at Asn20. A stick model of an LCAT allosteric activator is also shown bound spanning the interface between LCAT and HDL.  **e** The lid from a closed structure in yellow (PDB code 5TXF, with the rest of the lid modeled in), which swings out of the way (see arrow) when LCAT is activated by HDL. In all panels, side chains of crosslinked residues are shown as red spheres, and glycosylation sites as purple spheres for the first sugar. ApoA-I helix 6 is shown in cyan.

The proposed LCAT–HDL model not only accommodates the bulk of the experimental data and constraints, but also places LCAT near the helix 5–6 region of ApoA-I, which is proposed to be dynamic and have the potential to loop out (Fig. 4a–c)^30^. Such would provide easier access of LCAT to the lipid interior of the HDL particle. The multi-site reactivity of the lysines at the helix 5–6 junction is also consistent with highly dynamic behavior (Fig. 4b, Supplementary Table 2). The concave surface of LCAT in this orientation provides a path for phospholipid substrates from the HDL into the active site (assuming the ApoA-I helices loop out) that would allow hydrophobic lipids to interact with conserved hydrophobic residues on the interior of the concave surface of LCAT^9^ (Fig. 4d). It also allows space for the dynamic lid to be in a closed conformation and protect the active site until LCAT fully engages the HDL and opens into an active configuration (Fig. 4e). Furthermore, the docked model provides mechanistic hypotheses for how a small molecule activator that binds to the MBD may facilitate cholesterol esterification on HDL (Fig. 4d), one of which may be to help anchor the MBD into the HDL lipid bilayer in a way that optimizes the formation of a path for phospholipid transfer^6,33^. Thus, we propose that LCAT interacts initially with the lipid bilayer, likely via its hydrophobic N-terminal anchor, and that interactions with the amphipathic helices of ApoA-I facilitate MBD binding, active site lid opening, and stabilization of the active site.

We acknowledge that this is not the only possible model for the LCAT–HDL complex from our data. For example, it does not account for all the observed crosslinks, such as those involving LCAT-Lys240 and ApoA-I-Lys182. We know from the gel filtration profile of the complex, the 2D class averages, and the fact that all of the observed intermolecular crosslinks cannot be satisfied with a single docking model that this a heterogeneous system. To illustrate, in some 2D class averages the two LCATs have a greater angular spacing that would explain the crosslinks observed at ApoA-I-Lys182 and the nearby hotspot on helix 3 (Supplementary Fig. 2a). We also cannot predict where on the ApoA-I double belt a single subunit of LCAT would bind. It is possible therefore that LCAT can form favorable interactions with HDL at multiple sites that would account for the chemical crosslinking. In support of this idea, we have observed under some conditions negative stain 2D class averages wherein up to five LCAT enzymes are observed bound to one HDL particle (Supplementary Fig. 1b), although we do not expect more than one LCAT to be bound to HDL at a time under physiological conditions^38,39^.

There is also evidence for independent rotation of the two ApoA-I monomers leading to variable helical registries, such as a 5/2 and 5/4 orientation^19–22,31^. Some of our intermolecular ApoA-I crosslinks (Supplementary Table 1) suggest other registries wherein Lys77 and Lys88 can form intermolecular crosslinks, or additional ApoA-I monomers might create higher order oligomers as has been proposed as HDL matures^40^. Furthermore, a recent study examined the impact of the 5/5 and 5/2 helical registry on LCAT activity by locking different registries with disulfide bonds^19^. They identified that although LCAT can bind to HDL locked in either helical registry, only the 5/5 registry is able to activate LCAT to produce cholesteryl esters. This is consistent with previous work suggesting that LCAT binding to HDL is a separate step from LCAT activation by ApoA-I^5,26^. Thus, it appears that both helices of the double belt contact LCAT to create a productive site for LCAT activation, which our model supports. Additionally, as the 5/5 registry maximizes ApoA-I intermolecular salt bridges, this registry may be more competent to allow regions of the double belt to melt in order for substrate to reach LCAT. We can thus propose that LCAT is able to bind to the amphipathic helices of various apolipoproteins and different helical registries, but requires engagement to the helix 4/6 region in a 5/5 double belt for reactivity. However, we note that other apolipoproteins and amphipathic peptides are known to activate LCAT, some of which seem unlikely to be organized around the HDL in a similar manner to ApoA-I^41–43^. Thus, either LCAT reorganizes these helical elements or there are various modes of interaction that can drive more efficient cholesterol esterification.

Such questions would be facilitated by higher resolution information from LCAT bound to HDL, such as via cryo-electron microscopy. It will also be important to examine via EM and other biophysical methods what happens to the LCAT interaction as the HDL matures in the presence of cholesterol. The mechanism we propose with our docked model is dependent on access to the edge of a lipid bilayer in the discoid HDL particle, which suggests that as HDL particles mature LCAT will be able to bind, but increasingly lose its ability to form an energetically efficient pathway for lipids into its active site. In support of such a model, LCAT does appear to react with discs better than spheres; however, product inhibition by the cholesteryl ester could also explain this decreased reactivity^44^. Finally, examining HDLs made with other and/or additional apolipoproteins and in an oxidized state will help move towards visualizing the LCAT–HDL complex in a more physiological condition.

## Methods

### Protein expression and purification

LCAT protein used for the negative stain and XL-MS studies was received from MedImmune. As described previously, LCAT was purified from a stable cell line of CHO-S host cells using MedImmune’s proprietary fed-batch process in a bioreactor^5^. LCAT used for HDX-MS studies was purified as secreted recombinant protein from transiently transfected HEK293F (Invitrogen) cells as previously described^9^. The cells were grown in suspension and conditioned media was harvested 5 days later, then purified via Ni-NTA and dialyzed against reaction buffer (20 mM HEPES pH 7.5, 150 mM NaCl). Finally LCAT was purified via SEC with a Superdex 75 10/300 column (GE Healthcare) pre-equilibrated in reaction buffer. Mycoplasma testing was not performed, nor relevant as the resultant protein is the same regardless of mycoplasma status.

### Recombinant HDL preparation

A 3:1 lipid ratio of 1-palmitoyl-2-oleoyl-*sn*-glycero-3-phosphocholine (POPC) to 1,2-dipalmitoyl-sn-glycero-3-phosphoglycerol (DPPG) was used, with DPPG being incorporated to provide charge in order to prevent rouleaux formation. POPC and DPPG were dissolved in chloroform, and then dried under nitrogen flow at room temperature (RT) for 4 h before being placed in a vacuum oven overnight to remove residual chloroform. Tris/HCl buffer (20 mM Tris/HCl, 1 mM EDTA, and 0.02% NaN_3_, pH 8) was pre-heated to 55 °C, and then added to the lipid film to achieve a total lipid concentration of 16 µmol/mL, followed by vortexing (10 s) and water bath sonication (20 min, RT). The suspension was then mixed with 30 mg/mL sodium cholate in Tris/HCl buffer (0.737:1 molar ratio of lipid:sodium cholate) and heated in a 50 °C water bath for 1 min, followed by water bath sonication (10 min, RT) and probe sonication (3 W × 10 s, 18 times, room temperature) to obtain a translucent micelle solution. The micelle solution was mixed with ApoA-I at a 100:1 molar ratio of lipid:human ApoA-I purified from human serum^45^ which generates 9.6 nm HDLs. The mixture was gently shaken, then was incubated via thermal cycling between 50 and 0 °C until the solution was clear. Finally, the sodium cholate was removed by buffer exchange with a 10K Amicon Ultra 15 mL centrifugal filter (Merck Millipore) using 20 mM Tris/HCl, pH 7.5 as a wash buffer.

### Negative stain EM

The LCAT–HDL complex was prepared by pre-heating LCAT and HDL separately at 37 °C for 5 min and then together for 3 min at 37 °C. Because wild-type LCAT could hydrolyze the lipids even in the absence of cholesterol, a short incubation time was used. For complex formation, HDL at ~10 μM and LCAT at 120 µM in a total of 100 μL was injected onto tandem Superdex 200 10/300 columns (GE Healthcare) pre-equilibrated with 20 mM HEPES, 150 mM NaCl, 1 mM EDTA, pH 8. Samples were stained with 0.075% w/v uranyl formate^46^ and imaged with a Tecnai T12 electron microscope operated at 120 kV using low-dose procedures. Images were recorded at a magnification of ×71,138 and a defocus value of ∼1.4 μm on a Gatan US4000 CCD camera. All images were binned (2 × 2 pixels) to obtain a pixel size of 4.16 Å at the specimen level. A total of 20,295 particle projections were excised using Boxer (part of the EMAN 2.1 software suite)^47^ from 108 micrographs. Two-dimensional reference-free alignment and classification of particle projections were performed using iterative stable alignment and clustering (ISAC)^48^. In total, 16,421 projections of LCAT–HDL complexes were subjected to ISAC, producing 178 classes consistent over two-way matching and accounting for 8507 particle projections. 2D classification was also performed in Relion with 100 classes and some classes were observed to contain more than two LCATs, though a minor fraction (~1% of the particles) (Supplementary Fig. 1b). An ab initio model was generated with VIPER^28^ then used as an initial reference for maximum-likelihood-based 3D classification of 11,969 particles in RELION^29^. Two stable classes corresponding to the 1:1 and 2:1 LCAT:HDL models were generated from the 3D classification and then refined. The final 1:1 model was generated from 2641 particles, and the 2:1 model from 2913 particles. The 2:1 model was also refined with C2 symmetry and used for model building (Fig. 4), which is very similar to the C1 model shown in Fig. 1d. The C2 models with either possible hand were analyzed and fit the LCAT–HDL complex similarly, but we only show the model that positions the active site in a better orientation for catalysis, fits the inter-LCAT Lys105-Lys105 crosslink, and agrees with other published biochemical data.

### LCAT–HDL complex XL-MS

HDL (10 mg/mL stock) and LCAT (0.5 mg/mL stock) were diluted to 0.25 mg/mL (100 µL each) using 100 mM HEPES pH 7.4. Diluted HDL and LCAT were pre-heated separately for 5 min in a 37 °C water bath. Then, each component was combined to establish an interaction for 5 min at 37 °C. DC4 crosslinker (0.5 mg) was dissolved in 18.2 µL of 100 mM HEPES pH 7.4 and 10 µL of it was immediately added to the LCAT–HDL complex and placed in a 25 °C water bath. The mixture was incubated for 25 min and immediately injected onto an SEC column (TSKgel G3000SWXL 7.8 mm I.D. × 30 cm 5 µm) pre-equilibrated with PBS pH 7.4. The column was run with a flow rate of 0.25 mL/min, and fractions were collected every 1 mL starting at 18.4 ± 0.1 min. Individual fractions were dried under nitrogen at room temperature and stored at −20 °C. LCAT, HDL, and the LCAT–HDL complex controls were loaded separately to compare to the crosslinked complex_._

### XL-MS analysis

Prior to MS, the dried fractions containing the crosslinked LCAT–HDL complex were resuspended in 8 M urea, 20 mM HEPES (pH 8), and subsequently reduced using a final concentration of 4 mM DTT and alkylated by iodoacetamide at a final concentration of 20 mM. The fractions were digested overnight at 37 °C with two endopeptidases, GluC (Protea) and trypsin (Promega), in a 20:1 substrate to enzyme ratio. The peptide solutions were dried using a vacuum centrifuge and reconstituted in 0.1% trifluoracetic acid. Millipore C_18_ ZipTips were used to desalt the samples according to the manufacturer’s instructions. The samples were stored at −20 °C, after lyophilization, until MS analysis. The fractions were analyzed in the following way. For the first biological replicate, fractions 6 and 7 were kept separate and analyzed in duplicate in the mass spectrometer. For the second and the third biological replicates, fractions 6 and 7 were combined and then analyzed in the Lumos in triplicate (this is referred to as peak 1). Fraction 8 was analyzed a single time for each biological replicate (peak 2, Fig. 2b).

The crosslinked fractions were resuspended in 0.1% trifluoroacetic acid to a final concentration of 0.1 µg/µL and 1 µg was injected into an RP-HPLC Thermo Scientific Dionex UltiMate™ 3000 RSLC nanosystem coupled on-line with an Orbitrap Fusion Lumos Tribrid^TM^ (Lumos) mass spectrometer (Thermo Fischer Scientific, Inc., San Jose, CA, USA). The acquisition was performed for 120 min. Peptides were enriched on a pre-column, Acclaim PepMap 100 (C18, particle size 5 μm, 100 Å, 5 mm length, Thermo Fischer Scientific, Inc.), and separated on an analytical Acclaim PepMap 100 (C18, particle size 2 μm, 100 Å column; LC-Packings, Thermo Fisher Scientific, Inc.) of 50 cm bed length at a flow rate of 300 nL/min with a non-linear solvent gradient: 8 min, 98% A, and 2% B; 100 min, 45% B; 105 min, 90% B (A: water, 0.1% formic acid; B: 0.1% formic acid in 80% ACN).

The eluent was introduced into the mass spectrometer using a Nanospray Flex™ Ion Source. The MS was operated in positive ion and data-dependent mode with HCD-MS2-CID-MS3 as the acquisition strategy (Supplementary Fig. 4). Briefly, each selected MS1 precursor with charge state between 2 and 8 was subjected to stepped higher-energy collisional dissociation (HCD), 18–25–32% as energy values, with a precursor isolation width of 1 Da. Subsequently, mass-difference-dependent collision-induced dissociation (CID)-MS3 acquisitions were triggered if a unique mass difference (Δ = 112.1 Da, characteristic difference between DC4 signature peaks) was observed in the HCD-MS2 spectrum, and the normalized collision energy applied was 35% and the precursor isolation window was 1.6 *m*/*z* for signature peaks that have charge state between 2 and 5. Survey full scan MS1 (from *m/z* 375 to 2000) and MS2 were acquired in the Orbitrap with a respective mass resolution of 120,000 and 30,000, whereas MS3 scans were acquired in the ion trap. General MS conditions were electrospray voltage at 1.7 kV, no sheath and auxiliary gas flow, capillary temperature of 275 °C. Ion selection threshold was 400,000 counts for MS/MS, activation time of 50 ms.

### Crosslinked peptide analysis

The collected.raw files were directly analyzed using MS2MS3 analysis strategy of XLinkX node in Proteome Discoverer™ Software v2.2 (PD) or they were converted to.mzML files using ProteoWizard msConvert v3.0 with a peak peaking filter and then analyzed by MeroX v2.0 (ref. ^49^) (for examples see Supplementary Figs. 5–7). PD uses information from all MS levels, but only the lysine residue was used as site for DC4 modification. MeroX uses information only from MS1 and MS2, but lysine, serine, threonine, and tyrosine can be used as possible modification sites for DC4, as well as the N terminus. Predicted crosslinks to Ser residues were only reported when equivalent crosslinks to nearby Lys residues were also identified.

The files for peak 1 or peak 2 from biological and MS technical replicates were analyzed together in each software package. The setting for identification of crosslinked peptides was 5 ppm (PD) or 8 ppm (MeroX) mass tolerance for the precursor, and 15 ppm for fragment ions. Crosslinked peptides reported in this study had maximum XLinkX (PD) and MeroX scores corresponding to a false discovery rate (FDR)  < 0.02 and they were identified at least in two biological replicates across the two analyzed peaks.

### HDX-MS

LCAT–HDL complexes for HDX-MS were prepared by pre-heating LCAT and HDL separately at 37 °C for 5 min and then together for 3 min at 37 °C. HDL was at 13 μM and LCAT at 104 μM (1:8 ratio) in a total of 250 μL, which was then injected onto a Superdex 200 Increase 10/300 (GE Healthcare) pre-equilibrated with HDX buffer (10 mM HEPES, 150 mM NaCl, 1 mM EDTA, pH 8). Fractions corresponding to the LCAT–HDL complex were concentrated using a 50K Amicon Ultra 0.5 mL centrifugal filter (Merck Millipore) and kept on ice until analysis. Uncomplexed HDL alone was also injected on the Superdex column and concentrated similarly to the complex, whereas uncomplexed LCAT was diluted from the same stock as used for the complex into HDX buffer as it had already been purified via SEC.

HDX labeling data for uncomplexed LCAT, uncomplexed HDL, and the complex were collected at five time points (10 s, 30 s, 3 min, 10 min, 30 min), along with two undeuterated controls for each sample. See Supplementary Table 3 for more experimental details^50^. Sample concentrations for analysis were as follows: LCAT 20 μM, HDL 20 μM, and approximately 36 μM LCAT–HDL complex in equilibration buffer (10 mM HEPES, 150 mM NaCl, pH 8.0, H_2_O). For each labeling time, 3.0 μL of sample were diluted 15-fold (45 μL) with labeling buffer. The exchange reaction was allowed to proceed for each labeling time and labeling was quenched by the 1:1 (v:v) addition of ice-cold quench buffer (4.0 M GdnHCl, 250 mM TCEP, 150 mM NaCl, pH 2.37) to drop the pH to 2.5, followed by immediate placement on ice. All of the post-labeling steps were performed on ice with pre-chilled solutions and Eppendorf tubes. Sodium cholate (100 mM) was immediately added to the quenched samples to solubilize the lipoproteins, releasing ApoA-I for digestion. After the addition of sodium cholate, 12.0 μL of immobilized pepsin^51,52^ was added to the solution and allowed to digest for 5 min. After digestion, pepsin beads were removed from the solution utilizing Corning® Costar® Spin-X® centrifuge tube filters via centrifugation (10,000 × *g* at 4 °C). The flow-through was immediately introduced into a Waters nanoACQUITY with HDX technology^53^. Peptides were desalted for 3 min using an Acquity UPLC BEH C18 1.7 μm trap. After desalting, flow was reversed for chromatographic separation on an ACQUITY UPLC® HSS T3 1.8 μM, 1.0 × 50 mm analytical column. Peptides were eluted during a 20 min gradient, 5–35% water:acetonitrile 0.1% formic acid, flowing a 100 μL/min. Electrospray mass spectra were collected with a Waters Synapt G2Si operating in HDMS^E^ mode^54^. This procedure was repeated for each sample, at each time point, and for each replicate. Peptic peptides were identified with exact mass measurements and HDMS^E^ by ProteinLynx Global Server (PLGS) 3.0 software (Waters). Deuterium incorporation levels were determined using DynamX 3.0 software (Waters) along with the manual inspection of every spectrum to ensure accurate assignments of isotope clusters. The low variability in deuterium incorporation between the two independent manipulation replicates (0.12 Da LCAT; 0.18 Da ApoA-I) supports the reproducibility of our studies, and highlights the effects of LCAT binding to HDL^55^. For LCAT, we identified 115 peptides with an overall 75.2% coverage and 5.0 redundancy score (average number of overlapped peptides per amino acid residue; Supplementary Fig. 8). For ApoA-I we identified 101 peptides with 78.6% coverage and a 7.7 redundancy score (Supplementary Fig. 9).

### Statistics and reproducibility

For the negative stain dataset, only one sample was used for data collection, but analysis of other samples was similar. For XL-MS, three biological replicates were performed, with analysis performed with 1–3 technical repeats, as detailed above. The reported crosslinked peptides were defined by maximum XLinkX (PD) and MeroX scores corresponding to an FDR < 0.02 and being identified at least in two biological replicates across the two peaks. For the HDX-MS data, we performed two independent biological replicates with two technical replicates analyzed for each (Supplementary Table 3).

### Reporting summary

Further information on research design is available in the Nature Research Reporting Summary linked to this article.

## Supplementary information

Supplementary Information

Reporting Summary

Peer Review File

## Data Availability

The datasets generated during the current study are available from the corresponding author on reasonable request.
