# Supplementary material for: Structural analysis of lecithin:cholesterol acyltransferase bound to high density lipoprotein particles
Source: Commun Biol. 2020 Jan 15;3:28. doi: 10.1038/s42003-019-0749-z (PMC6962161; doi:10.1038/s42003-019-0749-z)
Supplement: Supplementary file 1 — Supplementary Information [file 42003_2019_749_MOESM1_ESM.pdf]

Supplementary Fig. 1: Negative stain EM data of the LCAT–HDL complex.

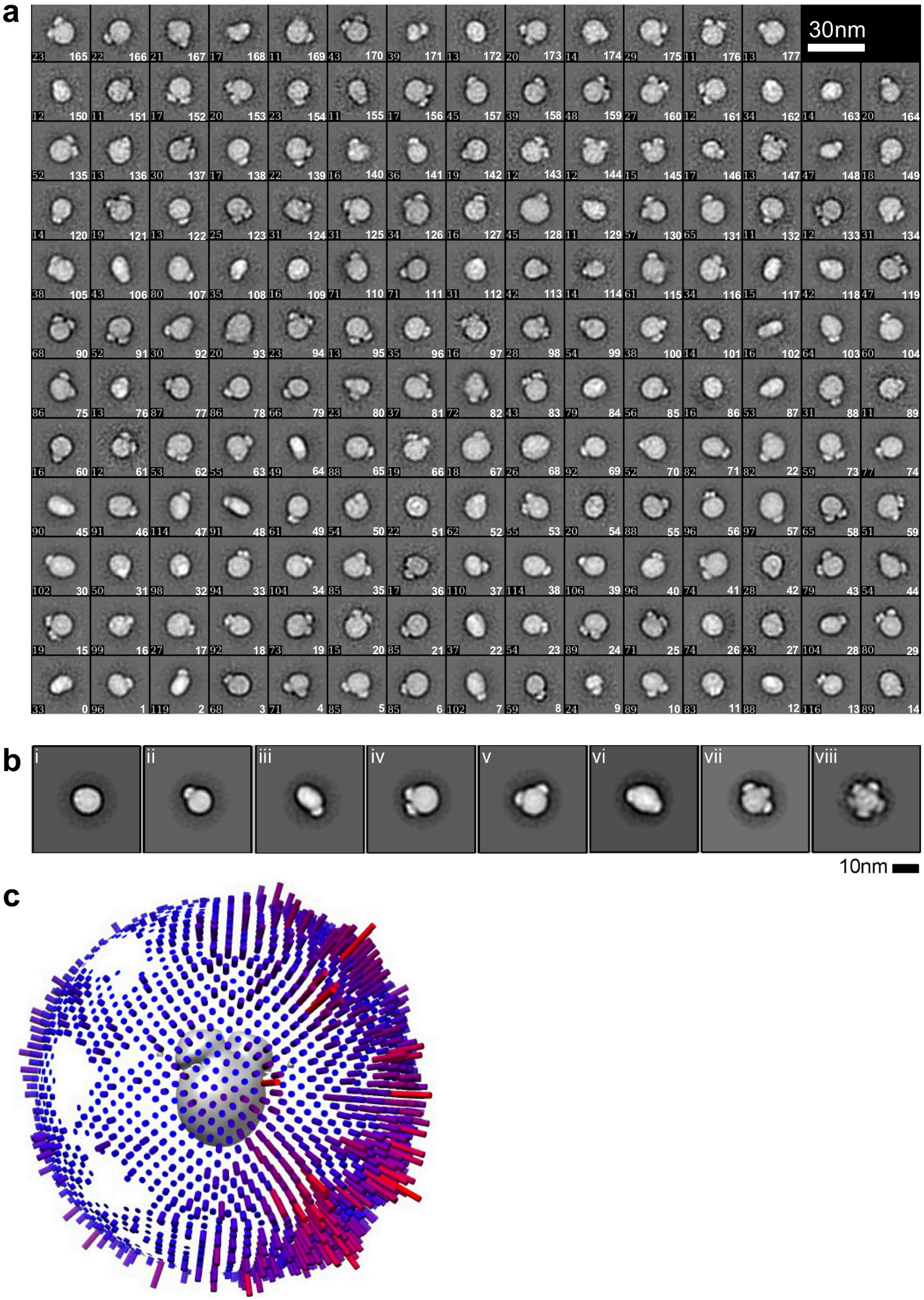

(**a**) 2D class averages generated from ISAC with 178 classes accounting for 8,507 particle projections. The number of particles in each class is shown in the bottom left and the class number in the bottom right. (**b**) Representative 2D class averages from Relion showing that up to five LCATs can bind to a single HDL particle. This heterogeneity helps to explain XL-MS results that are inconsistent with the most abundant assembly. (i) Isolated HDL particle (710 particles). (ii) One LCAT bound (555). (iii) Side/edge view of one LCAT bound (233) showing that it binds in a manner that is staggered with respect to the Apo-AI belt, as suggested by our 3D reconstructions (see Fig. 4a). (iv) Two LCAT bound with roughly 60˚ angular separation around the HDL disc (470). (v) Two LCATs bound, but this time with about 120˚ of separation (270). (vi) A side view of two LCAT bound with 120˚ of separation (233). (vii) Three LCATs bound (162), indicating that the enzyme has a propensity to bind at roughly 60˚ intervals around the circumference of the HDL. (viii) Five LCATs bound (66). The underlying message is that although typically only one LCAT would be expected to bind to an HDL *in vivo* and although there may be preferential binding to a particular Apo-AI region, the binding of LCAT to HDL is also somewhat nonspecific, indicating that context (amphipathic helices at the edge of the HDL lipid bilayer) is more important than sequence. However, activity may be highly dependent on sequence or the region of ApoA-I that is bound. (**c**) Angular distribution for the 2:1 LCAT:HDL 3D map showing the preferred orientation for the view normal to the plane of the HDL disc. Each cylinder represents an angular viewpoint, with the tallest red cylinder equal to the highest number of particles in that orientation.

Supplementary Fig. 2: The angular separation for two bound LCAT monomers is consistent with binding to helix 6 region of ApoA-I.

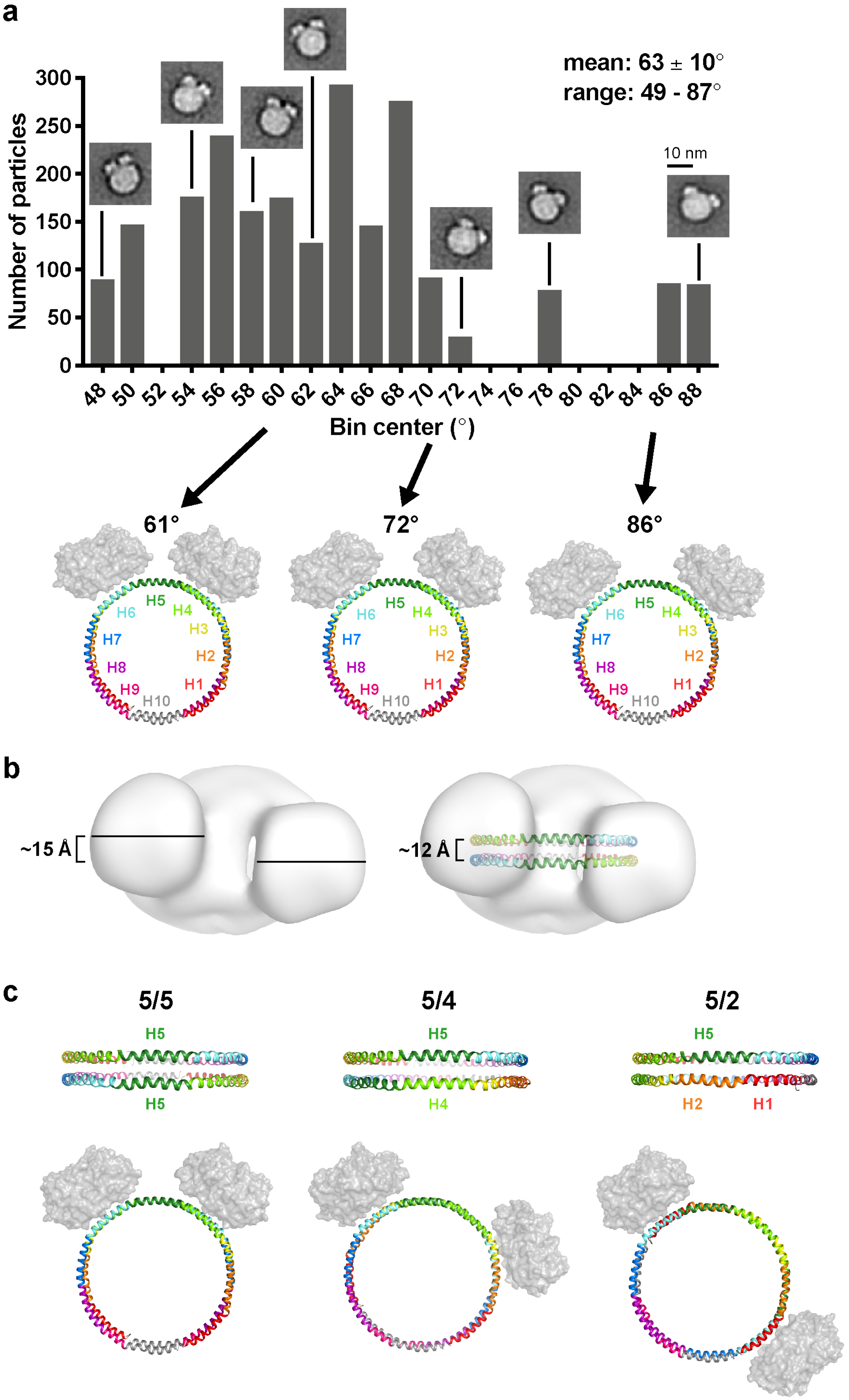

(**a**) The angle between the two LCATs in 2D class averages was measured using ImageJ[1]. The angles from 51 classes were then plotted as a histogram, with the number of particles in each class used to show the distribution in the dataset. Representative class averages are shown above the plot. Below, the arrows point to two models for how LCAT could bind to the ApoA-I belt to create the observed range of angles, with each binding at helix 6 (H6) or more towards H7. The ApoA-I monomers are each colored as a rainbow from H1 to H10 in the double belt model[2]. (**b**) The 3D reconstruction shows that two LCAT molecules bind to the side of the disc with an offset of ~15 Å along the central axis of the disc (left), which is consistent with them being bound to distinct ApoA-I chains, which are spaced ~12 Å apart in the double belt model (right). (**c**) The different registers of the ApoA-I double belt as proposed by the Davidson lab[3] would result in different binding modes for LCAT, here shown for example with LCAT bound to helix 6. 5/5 indicates H5 aligned with H5, etc. with the top showing the side view and the bottom image showing how LCAT would orient in a top-down view. The distribution observed in **a** is not consistent with alternative models presuming that LCAT preferentially binds to a specific helix (not necessarily H6).

Supplementary Fig. 3: Chromatographic analysis and 2D map of XL-MS results.

a

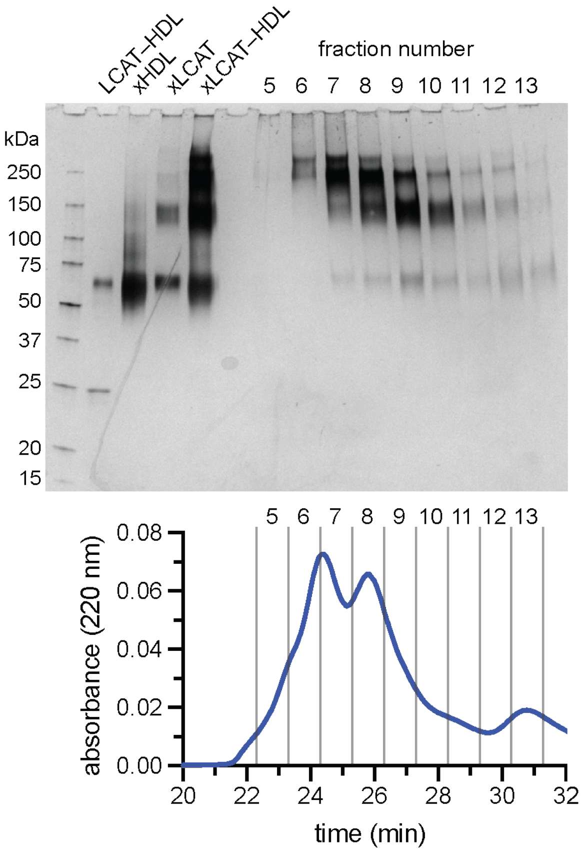

b

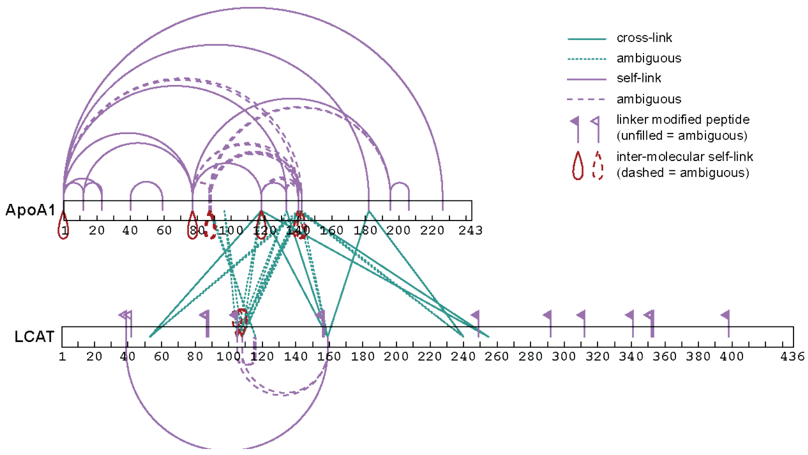

(**a**) 4-20% SDS-PAGE showing individual fractions from the crosslinked LCAT–HDL chromatogram (below). Fractions 6-7 were analyzed together (referred to as peak 1), and fraction 8 separately (referred to as peak 2). (**b**) Map of crosslinks identified within (purple) and between (green) LCAT and ApoA-I. The dashed lines refer to ambiguous crosslinks that occur within isolated peptides, such as the ambiguity between LCAT Lys105 and Ser108 (although Lys105 is more reactive).

Supplementary Fig. 4: Mass spectrometry workflow.

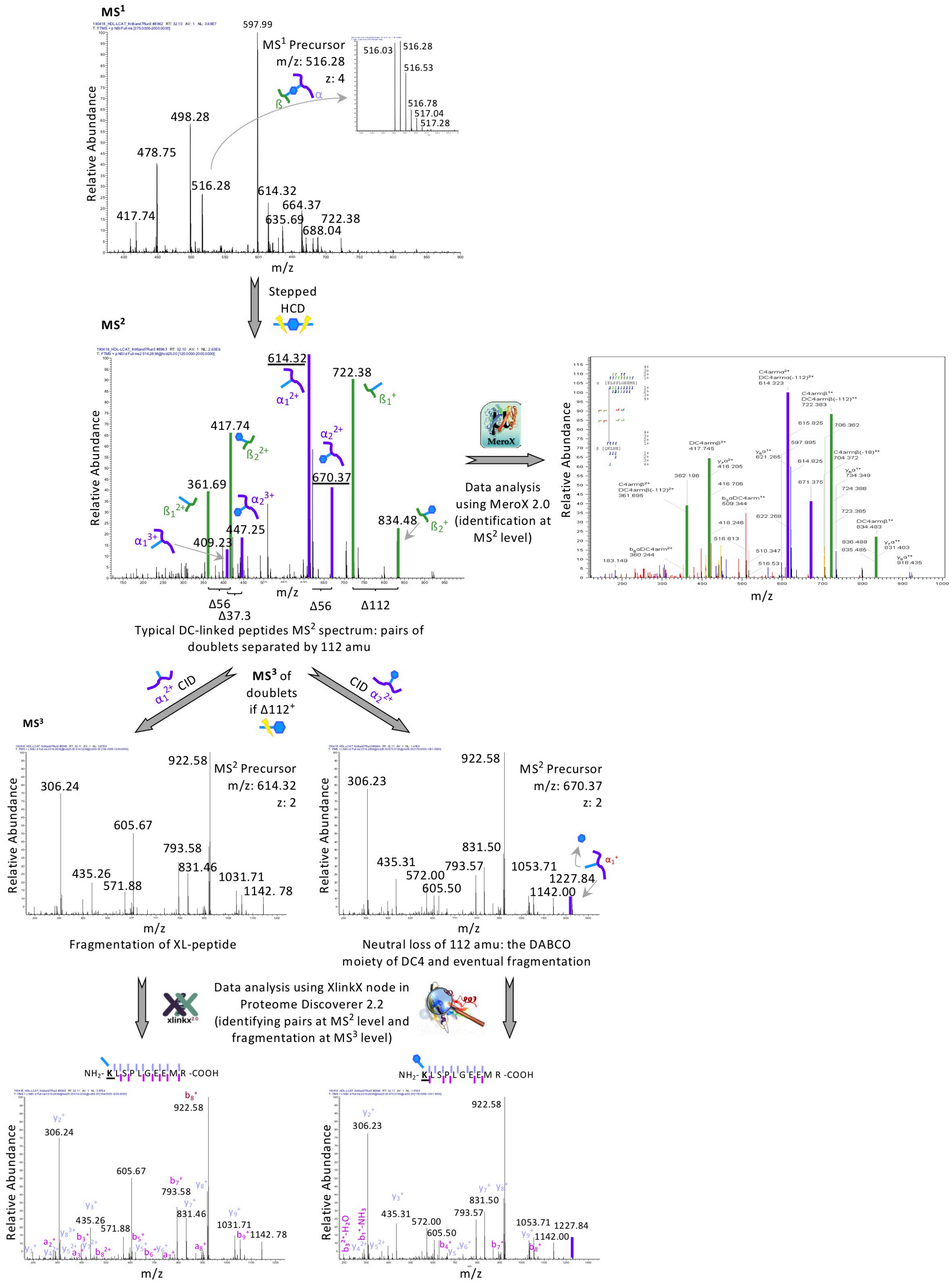

Crosslinks were identified using a mass spectrometry workflow that employed two separate programs: MeroX 2.0, which identifies crosslinks at the MS^2 level, and the XlinkX node in Proteome Discoverer 2.2, which identifies pairs at the MS^2 level and fragmentation at the MS^3 level.

Supplementary Fig. 5:Spectra showing identification of ApoA-I Lys140 crosslinked to LCAT Lys105/Ser108.

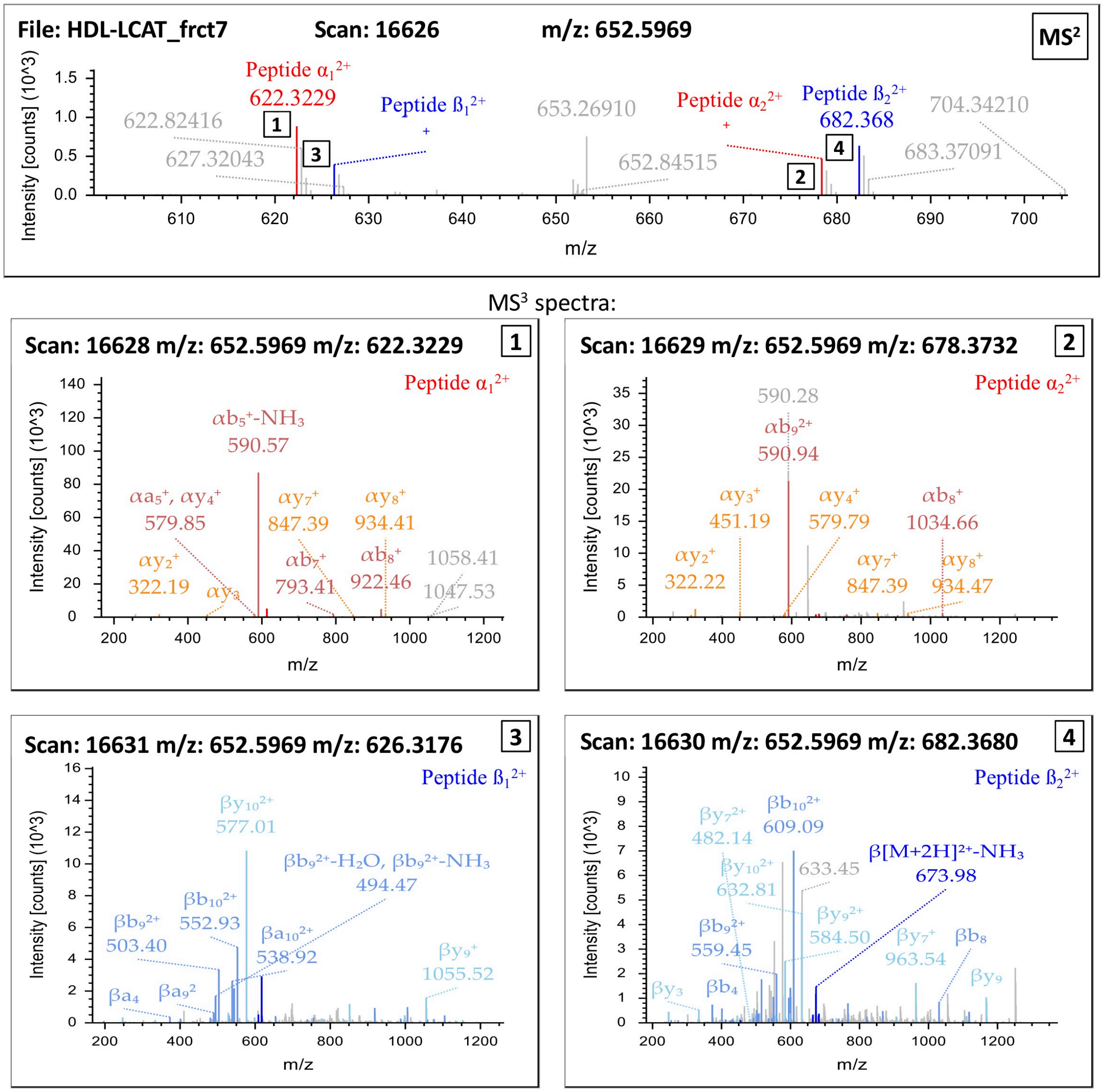

The spectra are from the XlinkX node of Proteome Discoverer 2.2, identifying pairs at the MS^2 level and fragmentation at the MS^3 level.

Supplementary Fig. 6: Spectrum identifying ApoA-I Lys140 crosslinked to LCAT Lys105/Ser108.

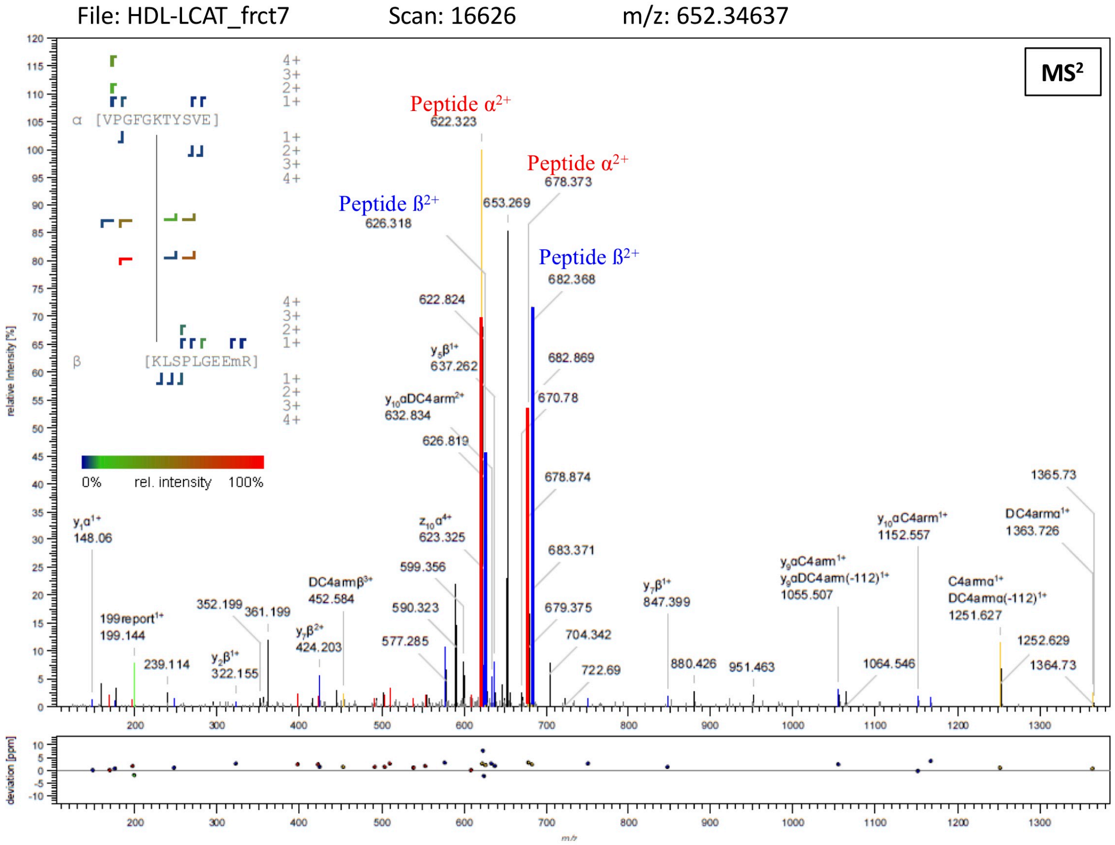

The spectrum is from MeroX identifying pairs at the MS^2 level.

Supplementary Fig. 7: Spectrum identifying intermolecular crosslinks between LCATs at Lys105/Ser108.

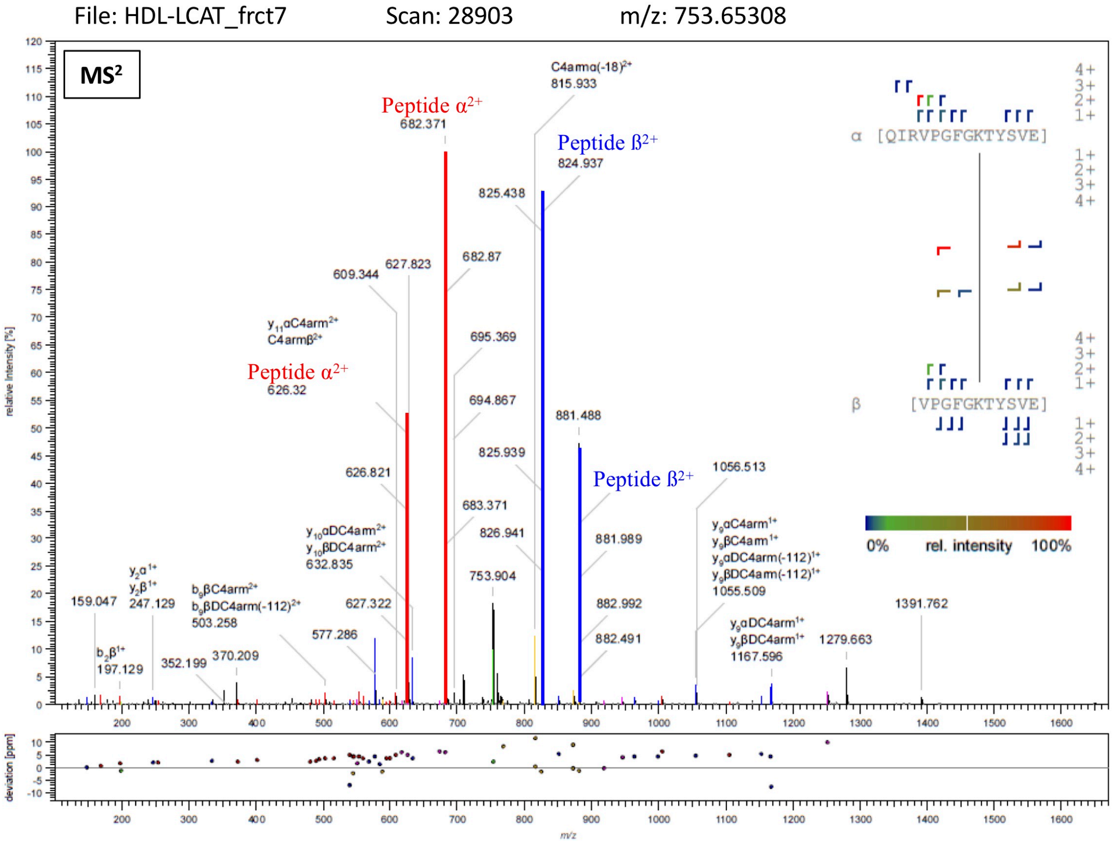

The spectrum is from MeroX identifying pairs at the MS^2 level.

Supplementary Fig. 8:HDX-MS LCAT peptide coverage map.

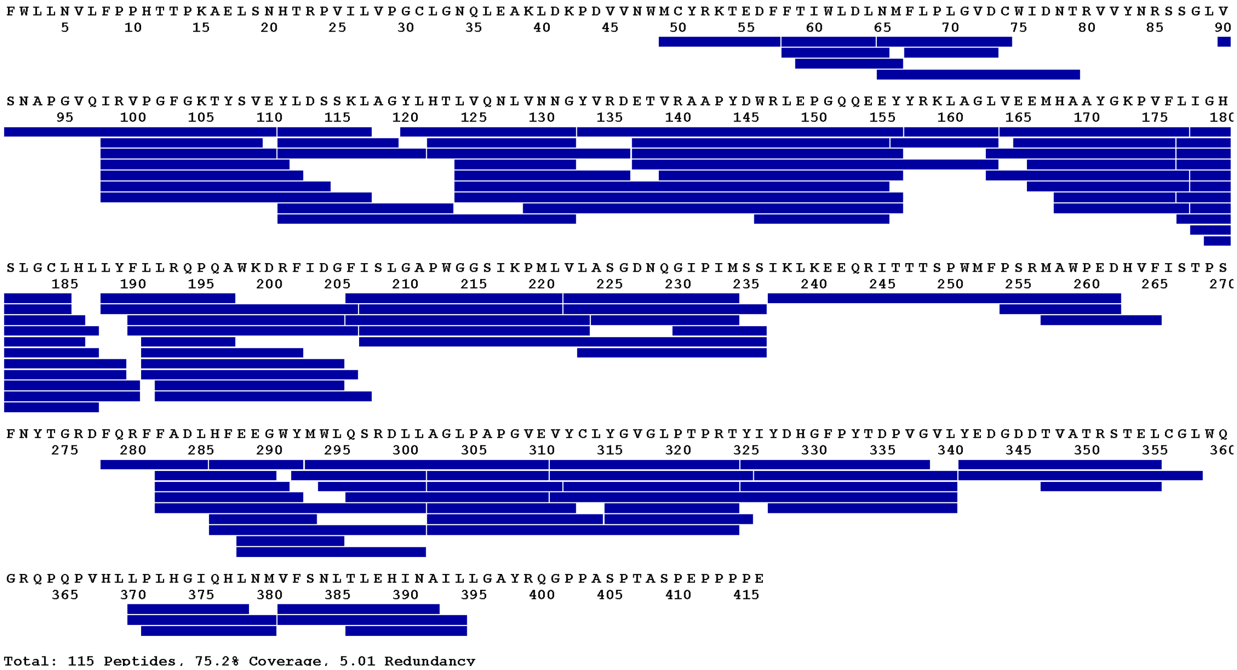

The amino acid sequence for human LCAT is shown at the top of each line. Dark blue bars represent peptic peptides identified and followed by HDX-MS for LCAT alone or HDL-bound.

Supplementary Fig. 9: HDX-MS ApoA-I peptide coverage map.

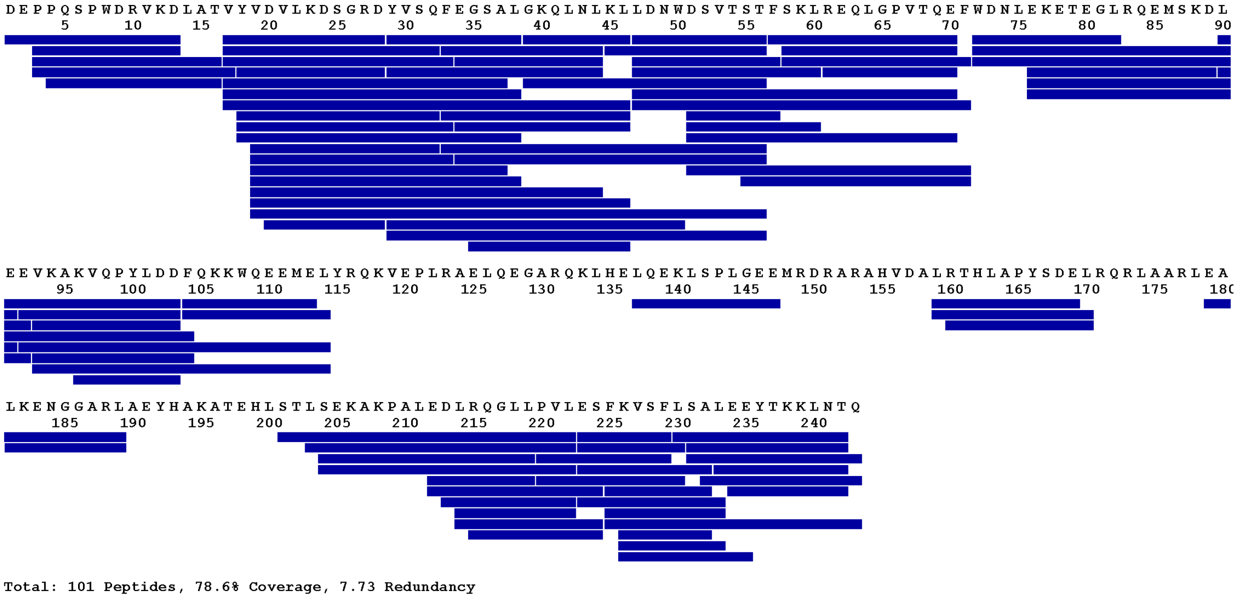

The amino acid sequence for human ApoA-I is shown at the top of each line. Dark blue bars represent peptic peptides identified and followed by HDX-MS for both ApoA-I in HDL alone and in HDL bound to LCAT.

Supplementary Fig. 10: Relative deuterium uptake curves for all peptides in the sequence coverage map.

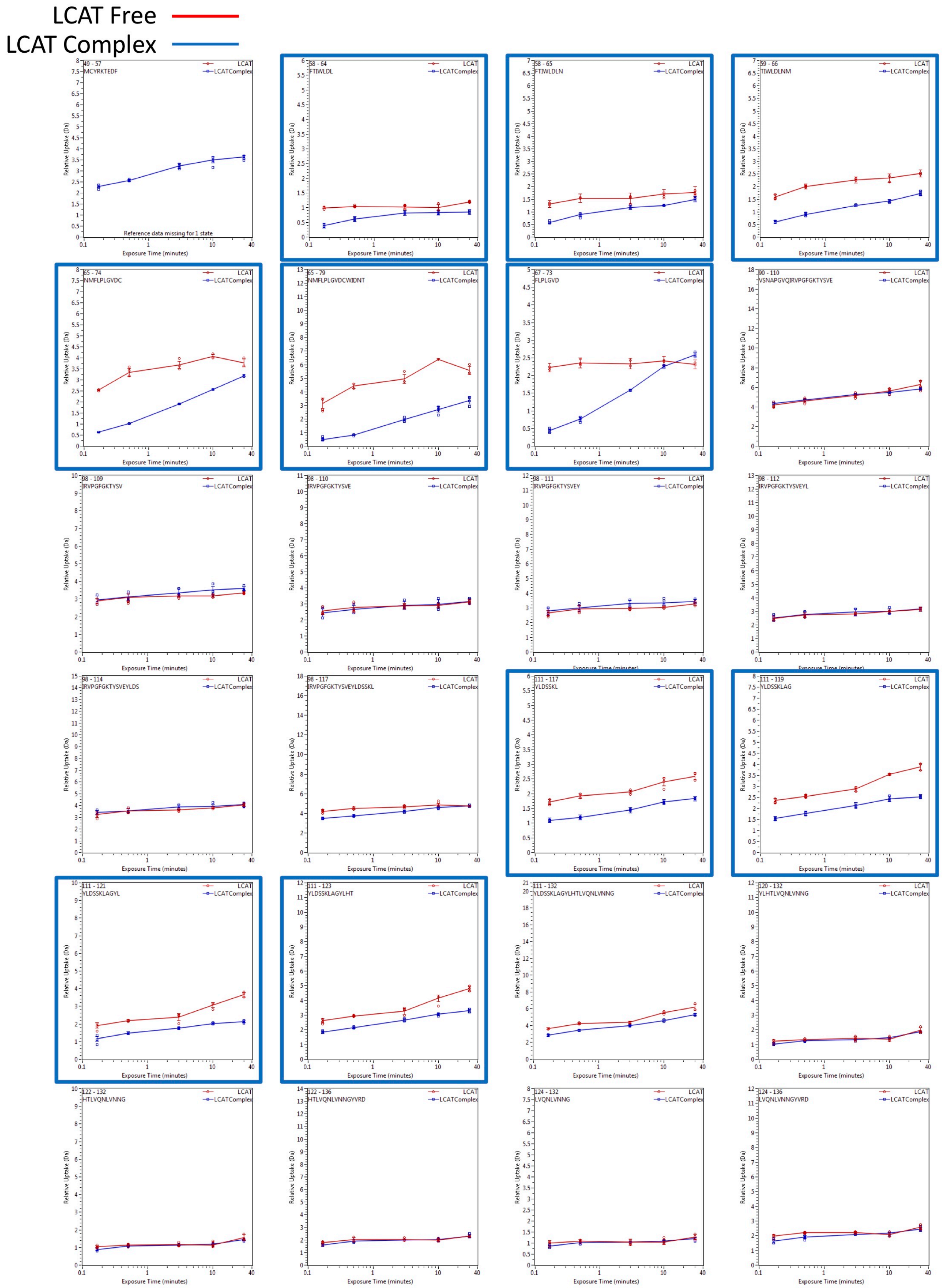

Supplementary Fig. 10 (cont)

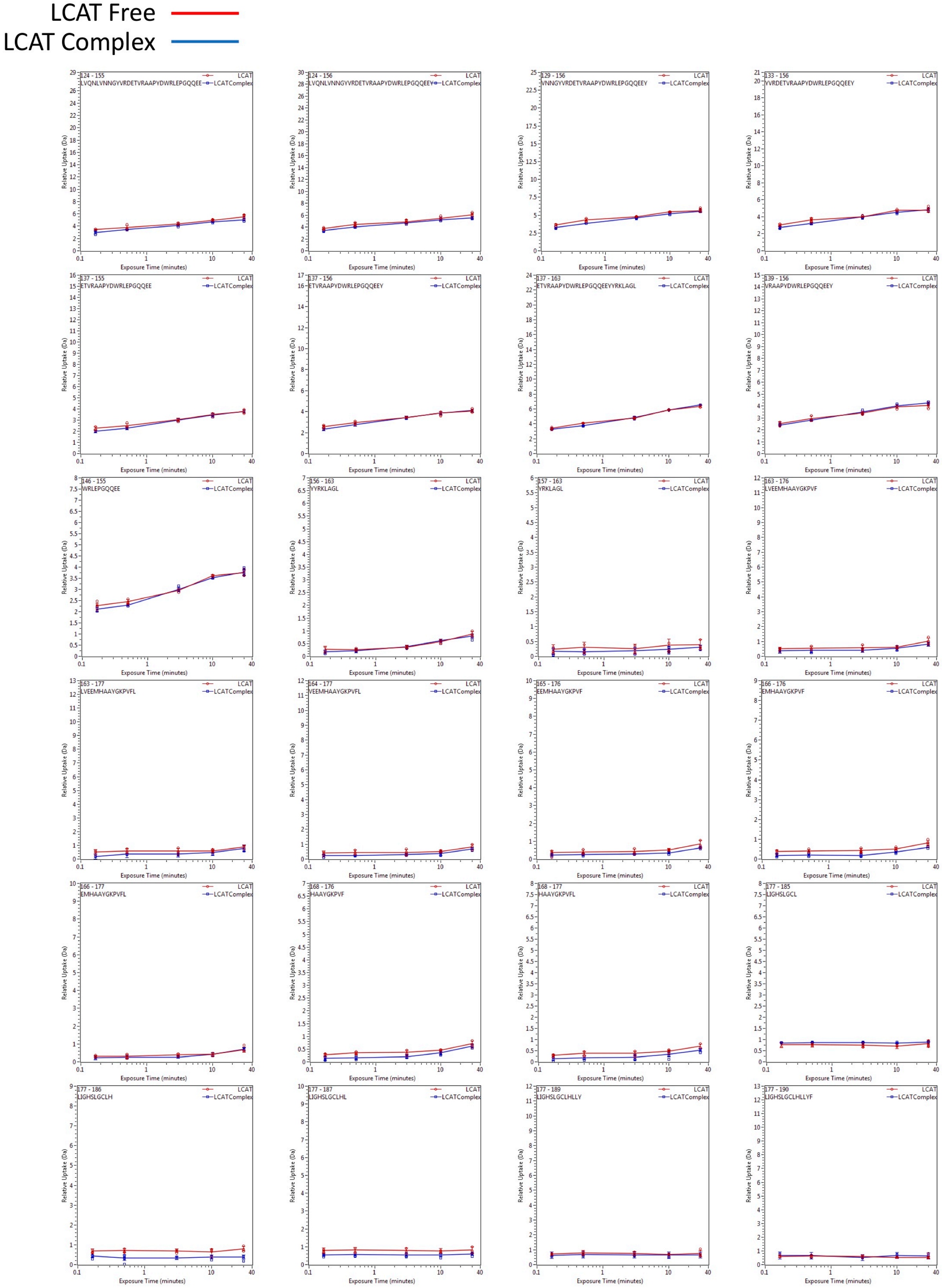

Supplementary Fig. 10 (cont)

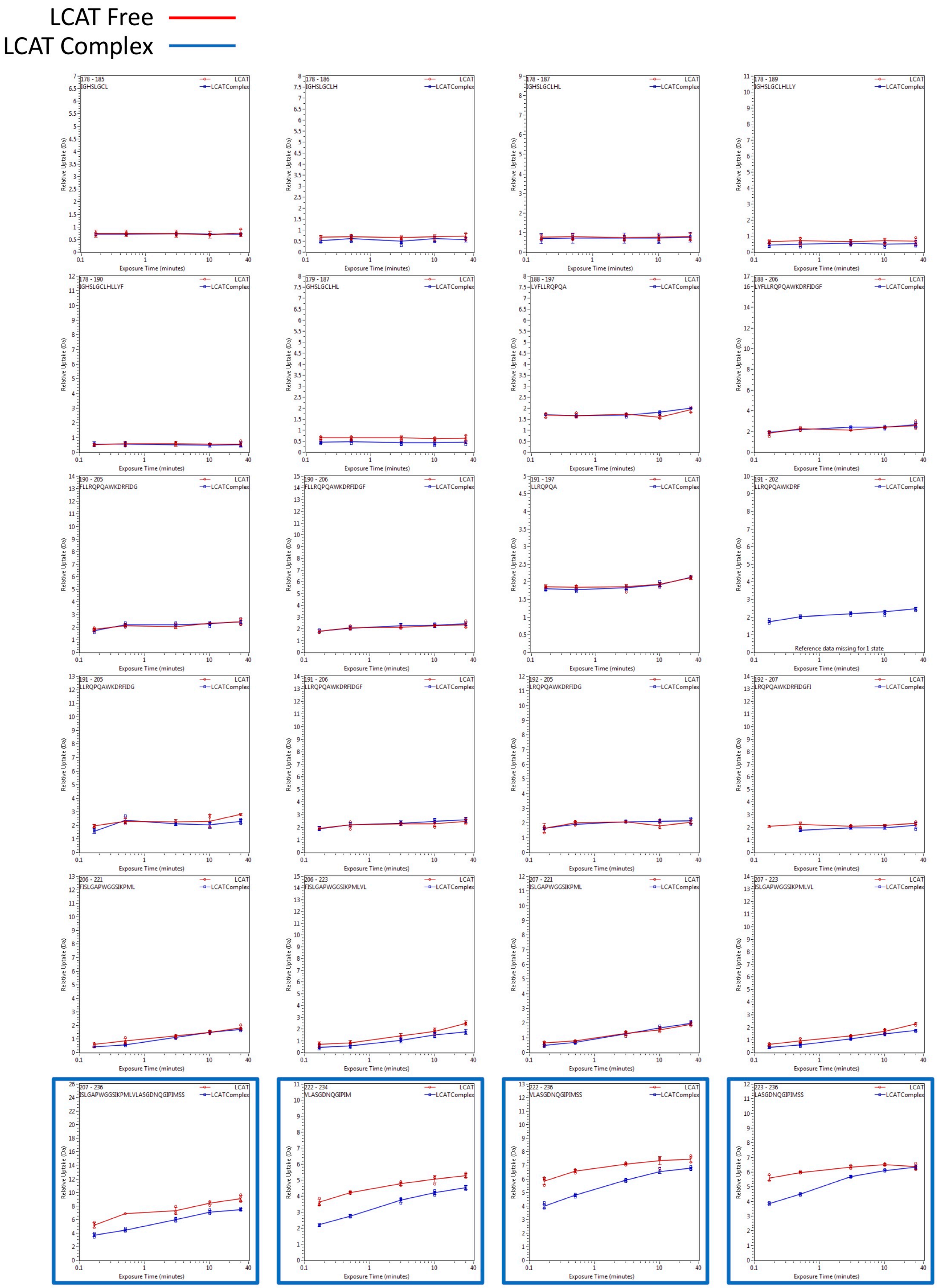

Supplementary Fig. 10 (cont)

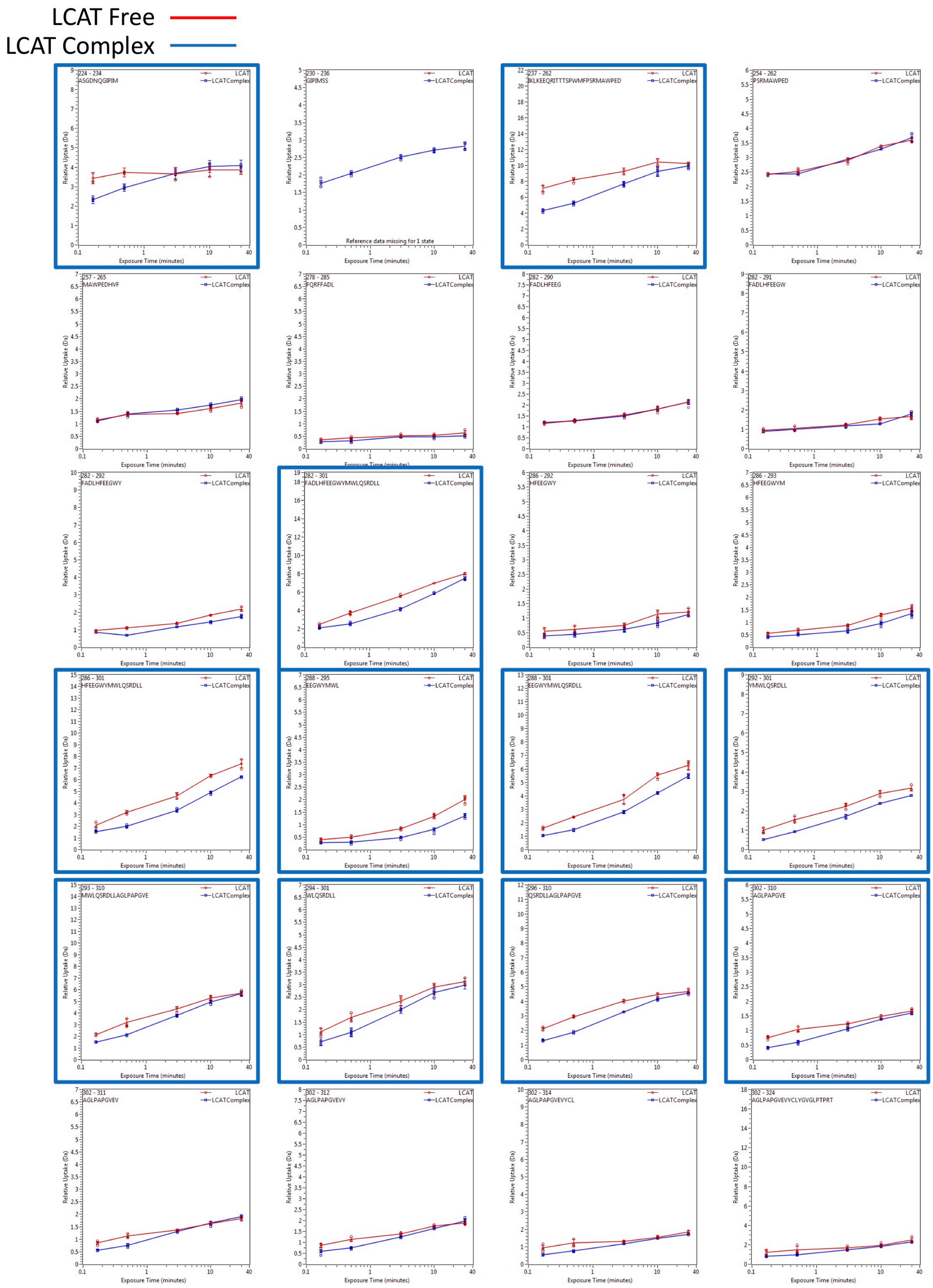

Supplementary Fig. 10 (cont)

LCAT Free
LCAT Complex

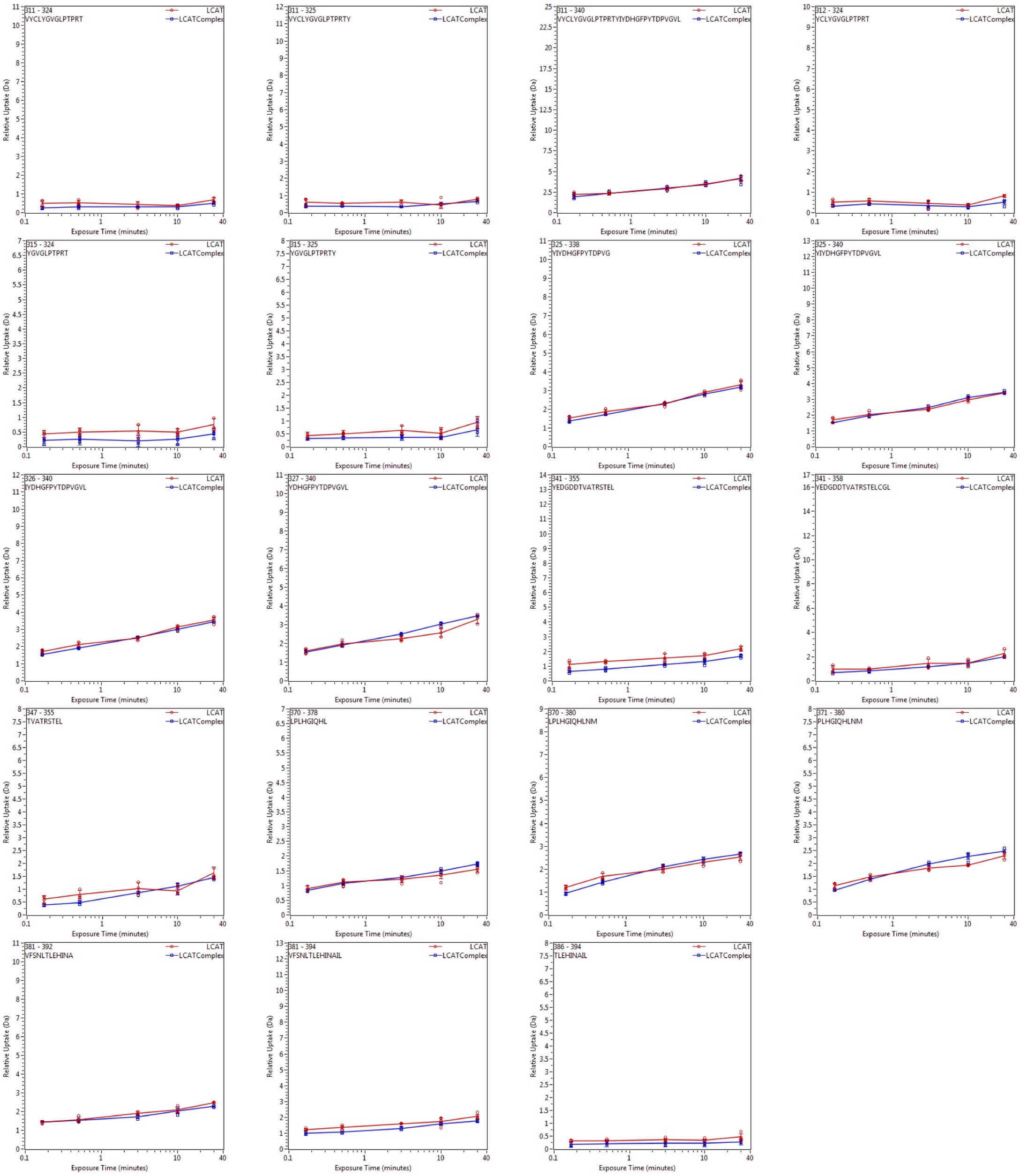

Protection Upon HDL Binding

Supplementary Fig. 10 (cont)

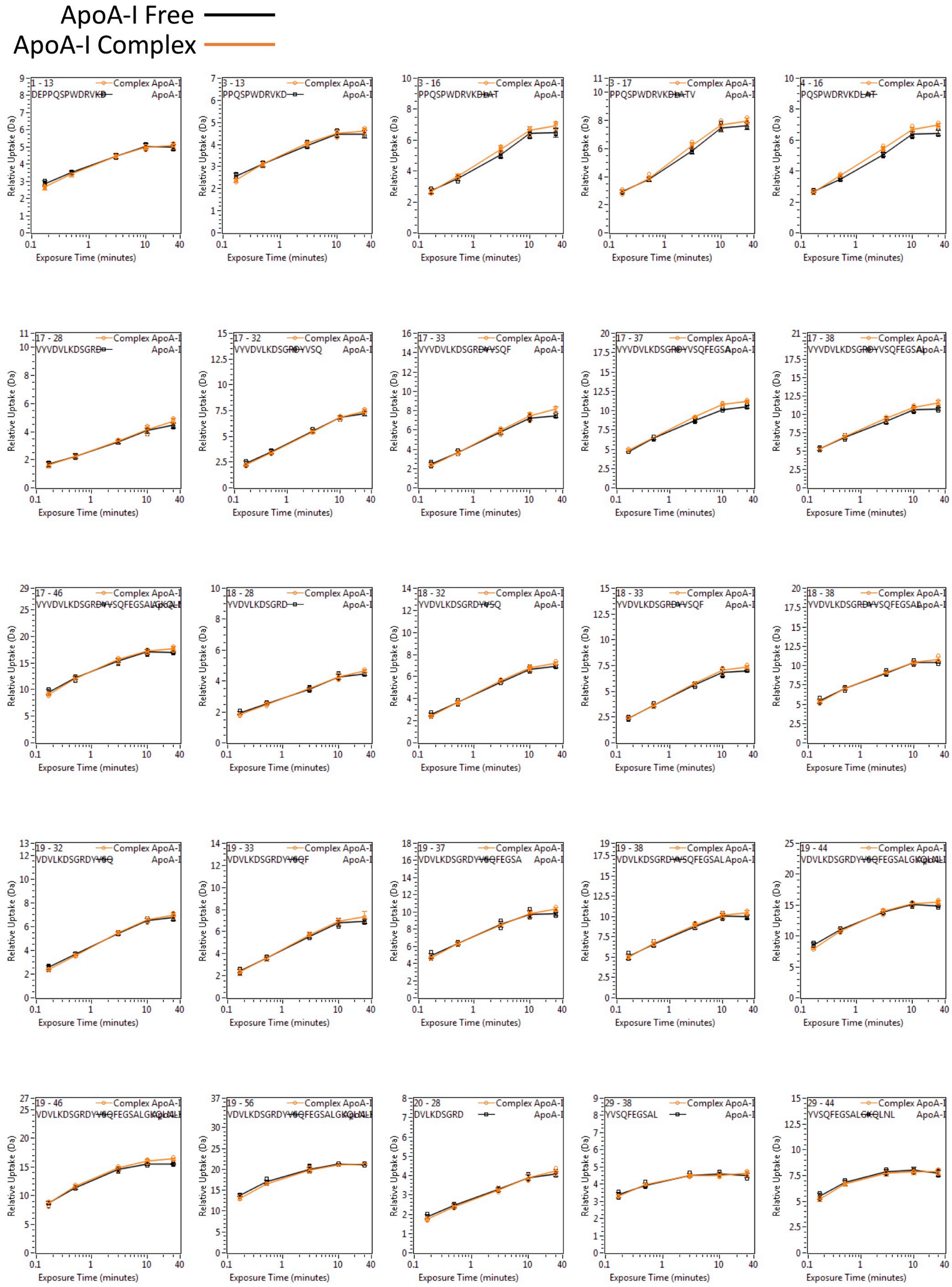

Supplementary Fig. 10 (cont)

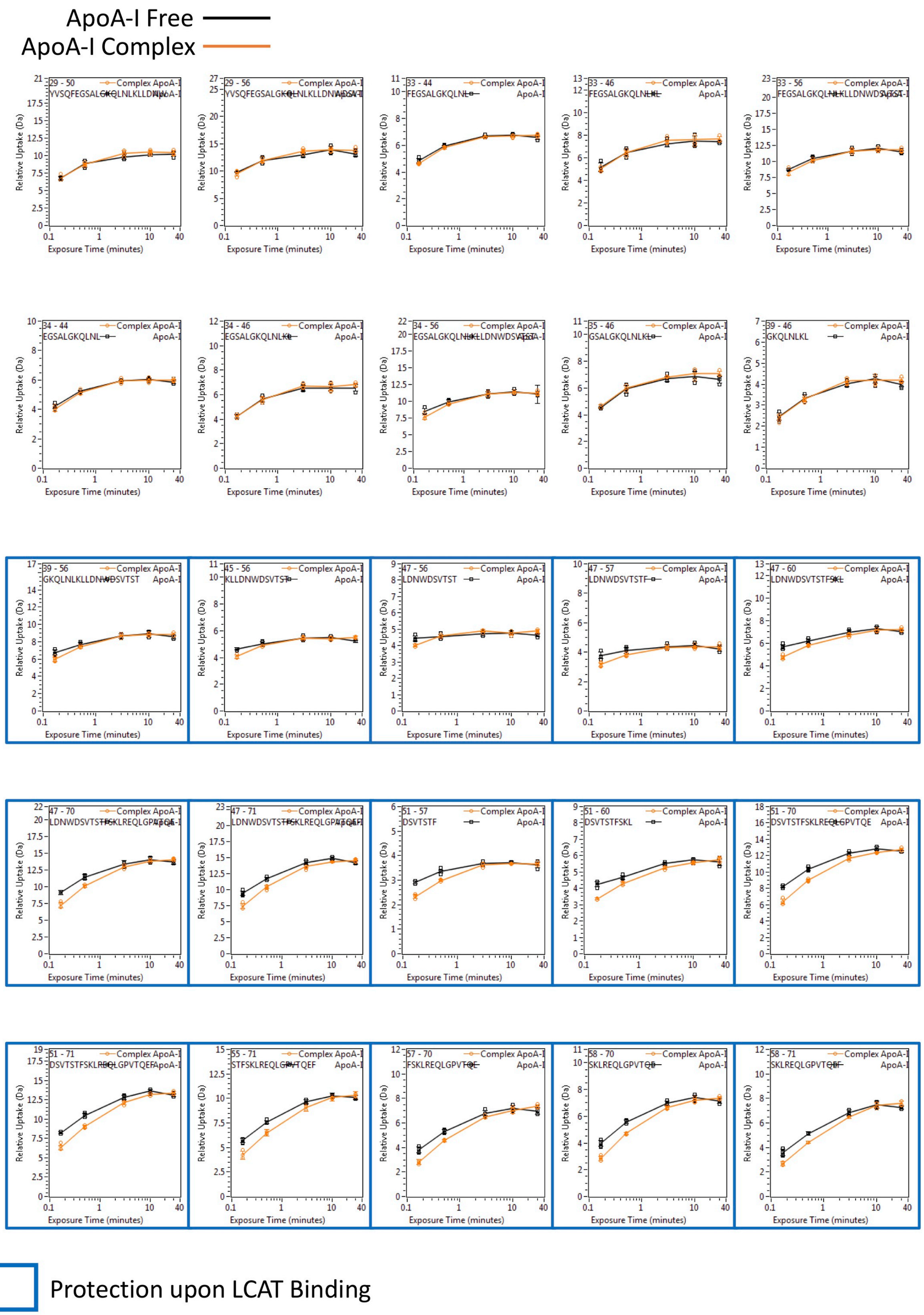

Supplementary Fig. 10 (cont)

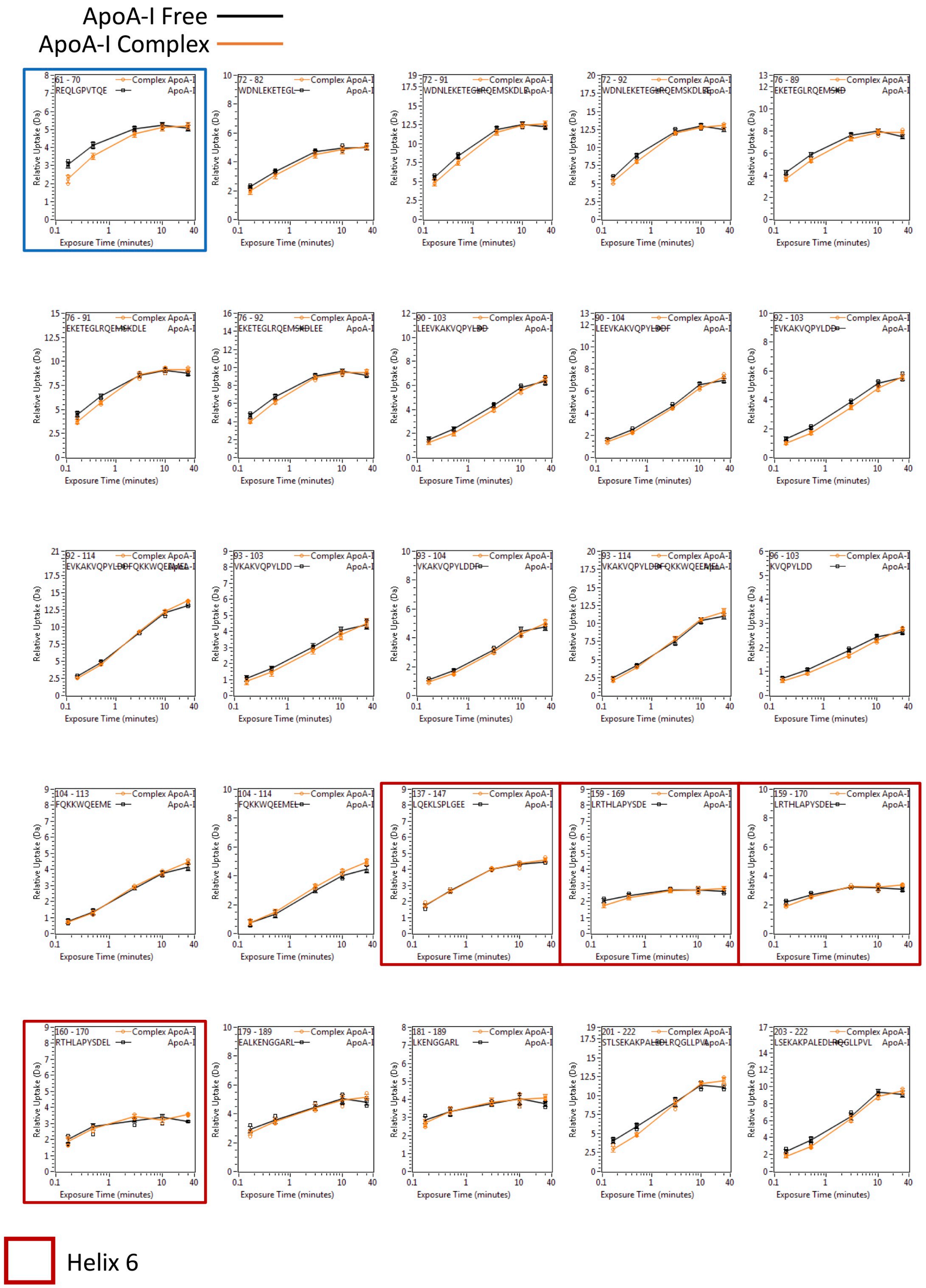

Supplementary Fig. 10 (cont)

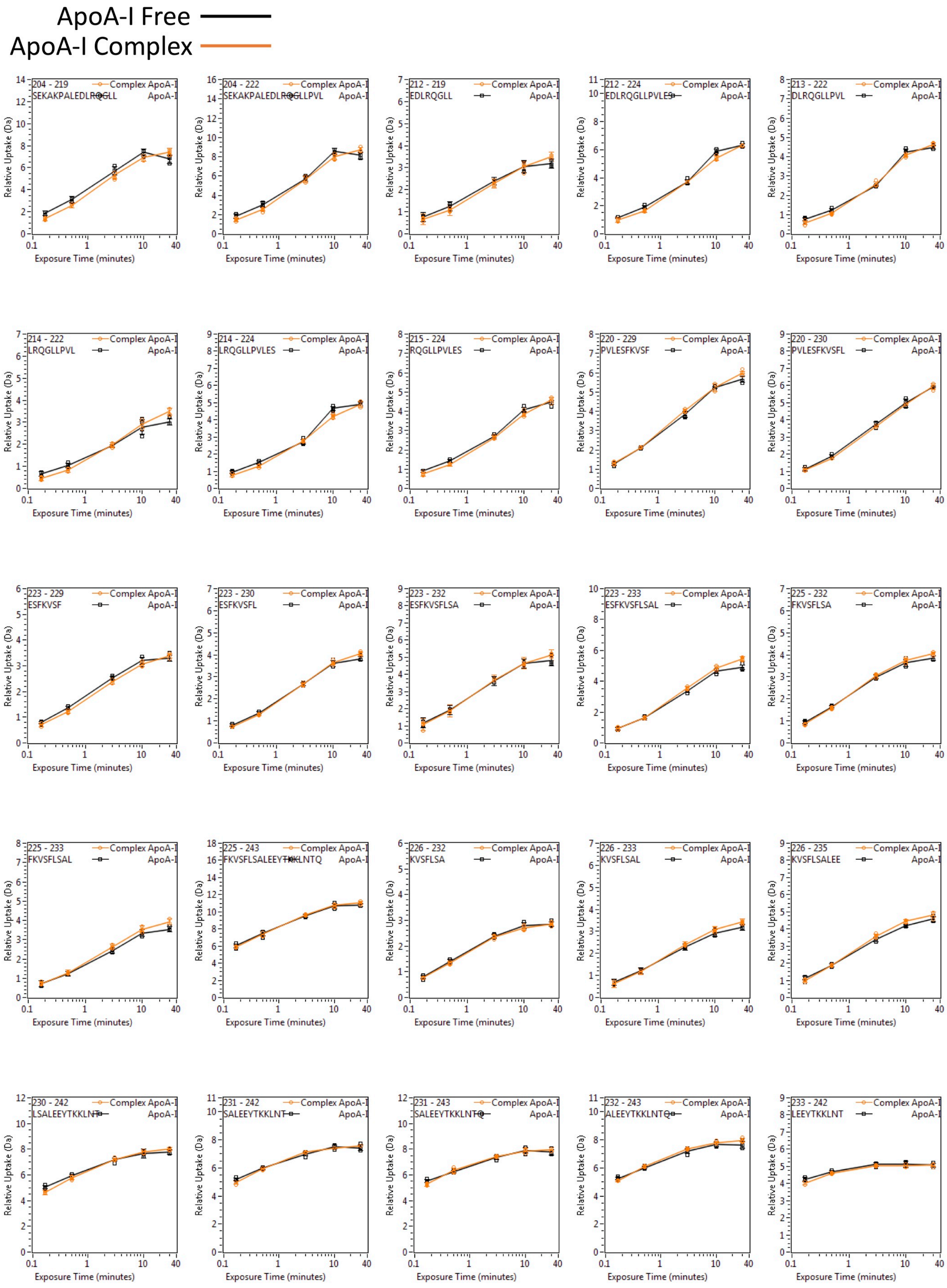

Supplementary Fig. 10 (cont)

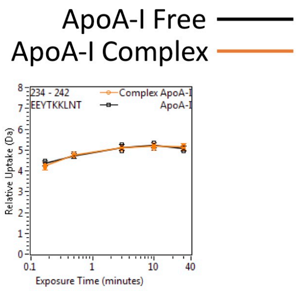

Free LCAT deuterium uptake (red) is compared to LCAT within the LCAT–HDL complex (blue) at 5 time-points, followed by free ApoA-I within HDL (black) compared to ApoA-I within the LCAT–HDL complex.

Supplementary Fig. 11: ApoA-I HDX data mapped onto the double belt HDL model.

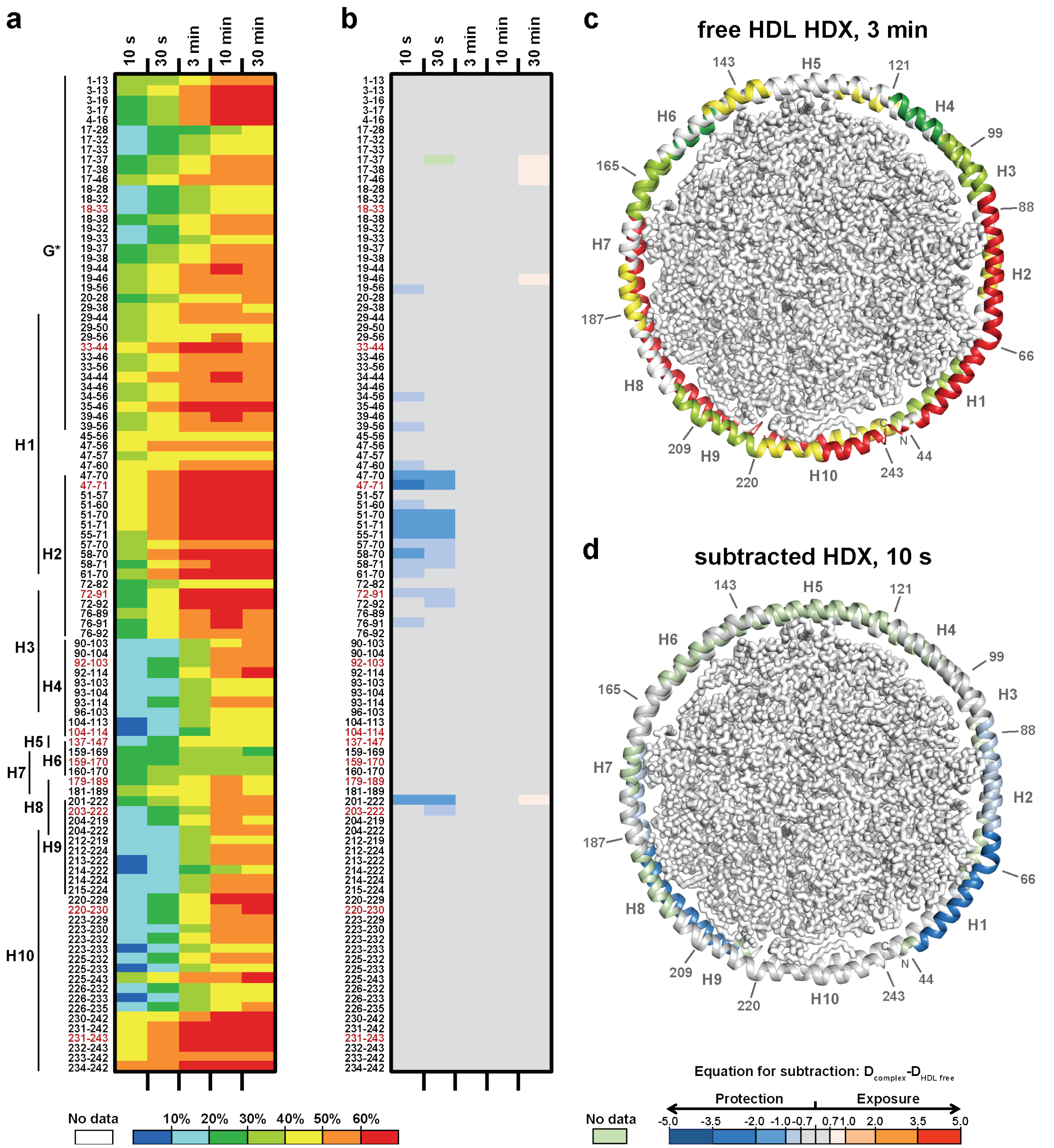

(**a**) The relative deuterium uptake for free HDL following ApoA-I peptides identified by HDX-MS, whereas (**b**) shows the difference ($D_{complex} - D_{HDL}$) between relative deuterium exchange in HDL when free and bound to LCAT. In both the exchange time is indicated at the top of the panel, increasing from 10 s to 30 min from left to right. The panels are color-coded according to the scales at the bottom, with free HDL uptake in increasing percentages (**a, c**) and the differences shown in Da (**b, d**). Down the left side are the residue numbers of each peptide fragment, arranged from N- to C- terminus (top to bottom). Peptides highlighted with red numbering are representative of the deuterium incorporation and were plotted onto the double belt model [2] in (**c**) for free HDL at the 3 min timepoint and (**d**) for the differences between free and LCAT-bound at the 10 s timepoint.

Supplementary Fig. 12: HDX data mapped onto open and closed LCAT structures.

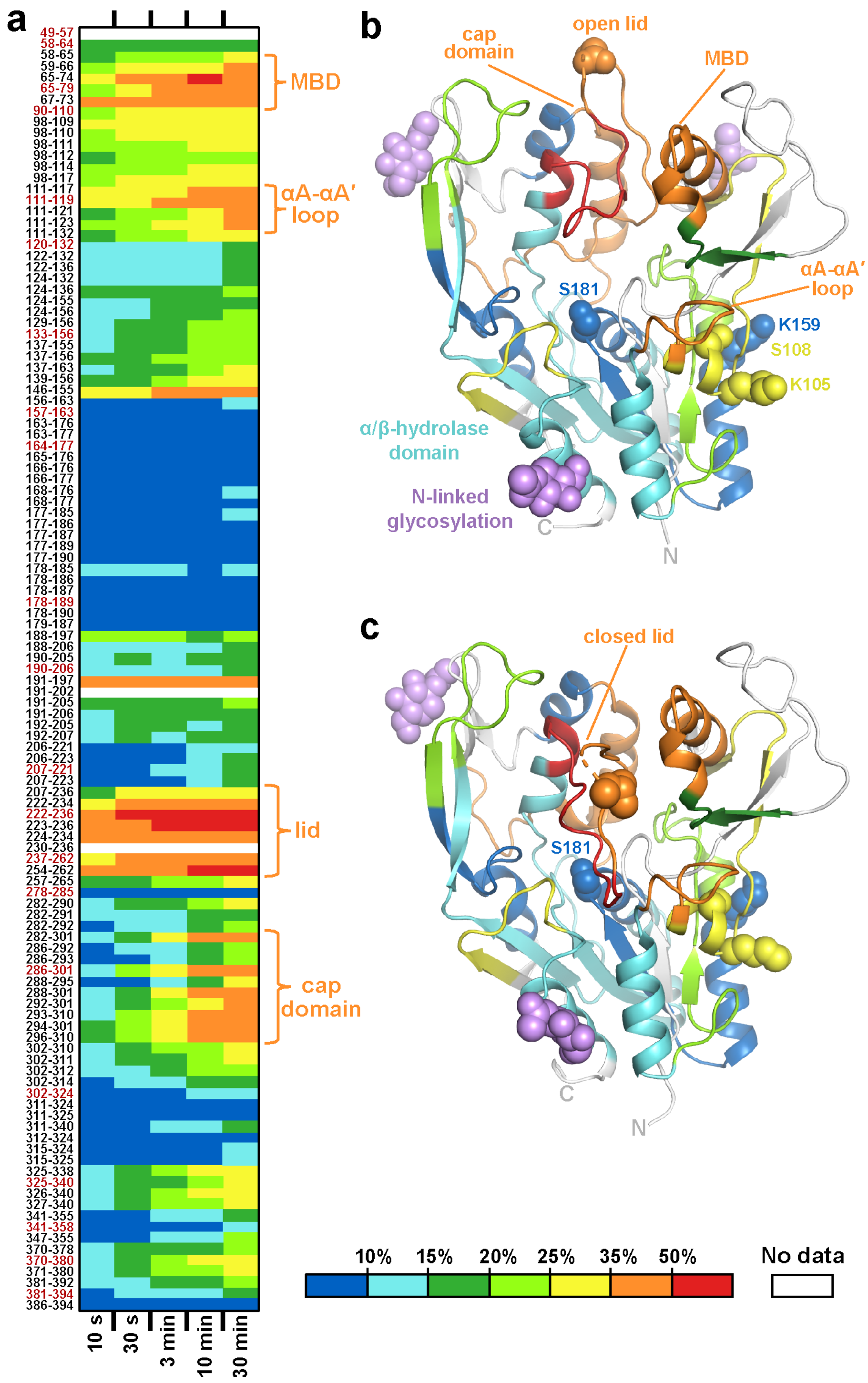

(**a**) The first panel shows the relative deuterium uptake for free LCAT for all peptides identified and followed by HDX-MS. The exchange time is indicated at the bottom of the panel, increasing from 10 s to 30 min from left to right. All differences are shown in Da and are color-coded according to the scale at the bottom. Down the left side are the residue numbers of each peptide fragment, arranged from N- to C- terminus (top to bottom). Peptides highlighted with red

numbering are representative of the differences in relative deuterium incorporation and were plotted onto two x-ray crystal structures of LCAT, (**b**) an open LCAT structure (PDB entry 6MVD) and (**c**) a closed lid conformation (PDB entry 5TXF) for the 10 min time point using PyMOL software.

Supplementary Table 1: Complete XL-MS data.

Crosslink		Crosslinked peptide sequence[a]		Peak 1[b]					Peak 2				
**LCAT**	**ApoA-I**	**LCAT**	**ApoA-I**	**Score[c]**		**CSM[d]**		**Rep[e]**	**Score**		**CSM**		**Rep**
K240	K140/S142	239-L**K**EEQR-244	137-LQE**K**L**S**PLGEEMR-149	263	148	27	8	3	213	133	6	2	2
K105/S108	K140/S142	100-VPGFG**K**TY**S**VE-110	137-LQE**K**L**S**PLGEEMR-149	203	91	16	8	3	206	35	1	2	1
K105/S108	K118	100-VPGFG**K**TY**S**VE-110	117-Q**K**VEPLRAE-125	183	142	10	3	2	153		1		1
K240	K182	239-L**K**EEQR-244	178-LEAL**K**ENGGAR-188	158		6		2	191		2		2
K105/S108	K94/K96	100-VPGFG**K**TY**S**VE-110	92-EV**K**A**K**VQPYLDDFQK-106	152		4		3	144	56	2	3	2
K105/S108	K133	100-VPGFG**K**TY**S**VE-110	132-Q**K**LHE-136	107	64	2	2	1	101	51	2	1	2
K159	K182	159-**K**LAGLVEE-166	178-LEAL**K**ENGGAR-188	105	53	3	2	2	55	57	2	2	2
K159	K140/S142	159-**K**LAGLVEE-166	137-LQE**K**L**S**PLGEEMR-149	92	172	16	13	3	42	139	2	1	1
K159	K133	159-**K**LAGLVEE-166	132-Q**K**LHE-136	37	107	5	1	3	38		2		2
K159	K118	159-**K**LAGLVEE-166	117-Q**K**VEPLRAELQEGAR-131	32	107	6	6	3					
K53	K140/S142	53-**K**TEDFFTI-60	137-LQE**K**L**S**PLGEEMR-149		143		14	1[e]		145		3	1
K53	K118	53-**K**TEDFFTI-60	117-Q**K**VEPLR-123		124		4	2					
S114/K116	S87/K88	111-YLD**S**S**K**LAGY-120	86-m**SK**DLEEVKA-95		115		23	2		111		6	2
S255	K140	252-MFP**S**R-256	137-LQE**K**LSPLGEEMR-149		64		109	2		64		42	2
S255	K118	252-MFP**S**R-256	117-Q**K**VEPLR-123		62		7	2		40		1	1
**LCAT**	**LCAT**	**LCAT**	**LCAT**	**Score**		**CSM**		**Rep**	**Score**		**CSM**		**Rep**
K105/S108	K159	100-VPGFG**K**TY**S**VE-110	159-**K**LAGLVEE-166	171	159	20	26	3	149	151	20	14	3
K105/S108	S114/S115/K116	100-VPGFG**K**TY**S**VE-110	111-YLD**SSK**LAGY-120	109	162	3	125	3	321	161	3	33	3
K39	K159	38-A**K**LDKPDVVNWMCYR-52	159-**K**LAGLVEE-166	51	112	8	2	2	26		3		3
K105/S108[f]	K105/S108	97-QIRVPGFG**K**TY**S**VE-110	100-VPGFG**K**TY**S**VE-110		139		14	3		145		8	3
**ApoA-I**	**ApoA-I**	**ApoA-I**	**ApoA-I**	**Score**		**CSM**		**Rep**	**Score**		**CSM**		**Rep**
K77	S87/K88	62-EQLGPVTQEFWDNLE**K**ETEGLRQE-85	79-TEGLRQEM**SK**DLEEVK-94	344	172	116	115	3	339	167	56	50	3
K77	K195	62-EQLGPVTQEFWDNLE**K**ETE-80	189-LAEYHA**K**ATEHLSTLSE-205	344	169	41	22	3	333	162	41	24	3
K133	K140/S142	132-Q**K**LHELQE-139	134-LHELQE**K**L**S**PLGEEMRDR-151	303	176	375	272	3	273	176	154	109	3
K140/S142[f]	K140/S142	134-LHELQE**K**L**S**PLGEEMRDR-151	140-**K**L**S**PLGEE-147	302	179	95	33	3	302	169	43	6	3
K118	K140/S142	117-Q**K**VEPLRAELQEGAR-131	137-LQE**K**L**S**PLGEEMRDR-151	292	157	290	100	3	314	153	127	38	3
K118	K133	117-Q**K**VEPLRAELQEGAR-131	132-Q**K**LHELQE-139	287	136	22	15	3	276	118	10	9	2
S87/K88	K195	79-TEGLRQEM**SK**DLEEVK-94	192-YHA**K**ATEHLSTLSE-205	259	117	43	13	3	258		13		2
K77	K118	62-EQLGPVTQEFWDNLE**K**E-78	117-Q**K**VEPLRAE-125	246	101	11	8	2	267	111	2	1	2
K12	K77	11-V**K**DLATVYVDVLK-24	71-FWDNLE**K**ETE-80	244	159	4	4	2	161	132	2	5	3
K118[f]	K118	117-Q**K**VEPLRAELQEGAR-131	117-Q**K**VEPLR-123	230	78	15	8	3	225	92	5	3	3
K195	K206	192-YHA**K**ATEHLSTLSE-205	206-**K**AKPALEDLR-215	211		10		2	124		3		1
K77[f]	K77	71-FWDNLE**K**E-78	74-NLE**K**ETE-81	169	96	2	7	2	150	70	1	3	1
S87/K88	K140/S142	84-QEM**SK**DLEEVK-94	140-**K**L**S**PLGEEMR-149	164	94	11	11	2	150	136	4	2	3
K77	K140/S142	71-FWDNLE**K**E-78	137-LQE**K**L**S**PLGEEMR-149	159	164	11	9	3	309	137	6	4	2
K88[f]	K88	78-ETEGLRQEMS**K**DLE-91	79-TEGLRQEMS**K**DLEE-92	81		2		2	158		2		2
K12	K23	11-V**K**DLATVYVD-20	21-VL**K**DSGR-27	53	126	4	14	3	37	125	1	5	3
Nt	K77	**{**DEPPQSPWDR-10	71-FWDNLE**K**E-78		153		35	3		150		19	3
Nt	K226	**{**DEPPQSPWDR-10	224-SF**K**VSFLSALEE-235		137		32	3		141		9	3
Nt[f]	Nt	**{**DEPPQSPWDR-10	**{**DEPPQSPWDR-10		130		17	3		138		6	3
Nt	K12	**{**DEPPQSPWDR-10	11-V**K**DLATVYVDVLK-23		126		12	2		133		12	3
Nt	K23	**{**DEPPQSPWDR-10	14-LATVYVDVL**K**DSGR-27		124		23	3		118		9	3
Nt	K140/S142	**{**DEPPQSPWDR-10	135-HELQE**K**L**S**PLGEEMRD-150		102		2	2					
Nt	K182	**{**DEPPQSPWDR-10	178-LEAL**K**E-183		78		10	3		146		6	3
Nt	K133	**{**DEPPQSPWDR-10	132-Q**K**LHE-136		63		6	3		68		1	1

[a]Peptide sequences with the highest score
[b]Each peak refers to the highlighted portion of the crosslinked SEC chromatogram in Figure 2b.
[c]The score and spectra in the left column are from Proteome Discoverer, and the right column from MeroX.
[d]Crosslink peptide-Spectra Matches.
[e]LCAT K53 ApoA-I K140 was found in only one replicate for each peak, but different replicates between the two peaks.
[f]Crosslinks between identical residues must be between two LCAT or ApoA-I proteins.
m: Oxidized methionine
Nt: ApoA-I N-terminus

Supplementary Table 2: Crosslinks that fit with the model shown in Fig. 4.

LCAT	ApoA-I	double belt	looped belt
**K240**	**K140**/S142	x	
**K105**/S108	**K140**/S142	x	x
**K105**/S108	**K118**	x	x
**K240**	**K182**		
**K105**/S108	K94/**K96**		
**K105**/S108	**K133**	x	x
**K159**	**K182**		
**K159**	**K140**/S142	x	x
**K159**	**K133**	x	x
**K159**	**K118**	x	x
**K53**	**K140**/S142	x	x
**K53**	**K118**	x	x
S114/**K116**	S87/**K88**		
**S255**	**K140**		
**S255**	**K118**		

LCAT	LCAT	double belt	looped belt
**K105**/S108	**K105**/S108	x	x

Bold residues indicate those used for measurements and a Cα-Cα distance cutoff of 40 Å was applied.

Supplementary Table 3: HDX-MS data summary and list of experimental parameters.

**Data Set**	Uncomplexed LCAT	Uncomplexed HDL	LCAT–HDL complex
**HDX reaction details**	Final D_2O concentration=93.3%, pH=8.0, 21 °C. See also footnote a		
**HDX time course**	10s, 30s, 3m, 10m, 30m		
**HDX controls**	2 undeuterated for each condition, 6 total		
**Back-exchange**	30-35%		
**Number of peptides**	Peptides in common between uncomplexed and complexed:<br>LCAT: 115; ApoA-I: 101		
**Sequence coverage**	LCAT: 75.2%; ApoA-I: 78.6%		
**Average peptide length**<br>**Redundancy**	Length – LCAT: 14.6 residues; ApoA-I: 13.6 residues<br>Redundancy – LCAT: 5.0; ApoA-I: 7.7		
**Replicates (technical)**	4 each: 2 technical replicates each of 2 independent complexes		
**Repeatability**	+/- 0.12-0.18 relative Da		
**Meaningful differences**	> 0.7 Da		

[a]15-fold dilution with labeling buffer [10 mM HEPES, 150 mM NaCl, pD 8.0, D_2O].1:1 v:v mix with quench buffer [4.0 M GdnHCl, 250 mM tris (2-carboxyethyl)phosphine hydrochloride (TCEP-HCl), 150 mM NaCl, pH 2.37, H_2O] to bring pH to 2.50

Supplementary References

1. Schneider, C.A., Rasband, W.S. & Eliceiri, K.W. NIH Image to ImageJ: 25 years of image analysis. *Nat Methods* **9**, 671-5 (2012).
2. Segrest, J.P. et al. A detailed molecular belt model for apolipoprotein A-I in discoidal high density lipoprotein. *J Biol Chem* **274**, 31755-8 (1999).
3. Silva, R.A., Hilliard, G.M., Li, L., Segrest, J.P. & Davidson, W.S. A mass spectrometric determination of the conformation of dimeric apolipoprotein A-I in discoidal high density lipoproteins. *Biochemistry* **44**, 8600-7 (2005).
