# Supplementary material for: Structural analysis of lecithin:cholesterol acyltransferase bound to high density lipoprotein particles
Source: Commun Biol. 2020 Jan 15;3:28. doi: 10.1038/s42003-019-0749-z (PMC6962161; doi:10.1038/s42003-019-0749-z)
Supplement: Supplementary file 2 — Reporting Summary [file 42003_2019_749_MOESM2_ESM.pdf]

nature research

Corresponding author(s): John J.G. Tesmer

Last updated by author(s): Dec 11, 2019

Reporting Summary

Nature Research wishes to improve the reproducibility of the work that we publish. This form provides structure for consistency and transparency in reporting. For further information on Nature Research policies, see Authors & Referees and the Editorial Policy Checklist.

Statistics

For all statistical analyses, confirm that the following items are present in the figure legend, table legend, main text, or Methods section.

n/a	Confirmed	
☐	☒	The exact sample size (*n*) for each experimental group/condition, given as a discrete number and unit of measurement
☐	☒	A statement on whether measurements were taken from distinct samples or whether the same sample was measured repeatedly
☒	☐	The statistical test(s) used AND whether they are one- or two-sided<br>*Only common tests should be described solely by name; describe more complex techniques in the Methods section.*
☒	☐	A description of all covariates tested
☒	☐	A description of any assumptions or corrections, such as tests of normality and adjustment for multiple comparisons
☒	☐	A full description of the statistical parameters including central tendency (e.g. means) or other basic estimates (e.g. regression coefficient) AND variation (e.g. standard deviation) or associated estimates of uncertainty (e.g. confidence intervals)
☒	☐	For null hypothesis testing, the test statistic (e.g. *F*, *t*, *r*) with confidence intervals, effect sizes, degrees of freedom and *P* value noted<br>*Give P values as exact values whenever suitable.*
☒	☐	For Bayesian analysis, information on the choice of priors and Markov chain Monte Carlo settings
☒	☐	For hierarchical and complex designs, identification of the appropriate level for tests and full reporting of outcomes
☒	☐	Estimates of effect sizes (e.g. Cohen's *d*, Pearson's *r*), indicating how they were calculated

Our web collection on statistics for biologists contains articles on many of the points above.

Software and code

Policy information about availability of computer code

Data collection	DigitalMicrograph (low dose mode), Xcalibur v4.0 (Thermo)
Data analysis	Boxer (EMAN 2.1 software suite), ISAC2 (in Sphire/EMAN2.2), VIPER, Relion 3.0.2, XLinkX node of Proteome Discoverer™ Software v2.2, ProteoWizard msConvert v3.0, MeroX v2.0, ProteinLynx Global Server (PLGS) 3.0 software (Waters), DynamX 3.0 software (Waters)

For manuscripts utilizing custom algorithms or software that are central to the research but not yet described in published literature, software must be made available to editors/reviewers. We strongly encourage code deposition in a community repository (e.g. GitHub). See the Nature Research guidelines for submitting code & software for further information.

Data

Policy information about availability of data

All manuscripts must include a data availability statement. This statement should provide the following information, where applicable:
- Accession codes, unique identifiers, or web links for publicly available datasets
- A list of figures that have associated raw data
- A description of any restrictions on data availability

The datasets generated during and/or analyzed during the current study are available from the corresponding author on reasonable request.

Field-specific reporting

Please select the one below that is the best fit for your research. If you are not sure, read the appropriate sections before making your selection.

☒ Life sciences ☐ Behavioural & social sciences ☐ Ecological, evolutionary & environmental sciences

Life sciences study design

All studies must disclose on these points even when the disclosure is negative.

Sample size	N/A
Data exclusions	For negative stain, classes were excluded that did not resemble HDL particles during class averaging. Crosslinked peptides reported in this study had maximum XLinkX (PD) and MeroX scores corresponding to FDR < 0.02 and they were identified at least in two biological replicates across both analyzed peaks.
Replication	For the negative stain data set, only one sample was used for data collection, but analysis of other samples looked similar. For crosslinking, three biological replicates were performed with 1-3 technical replicates as detailed in the methods. For HDX MS, there were 2 technical replicates each of 2 independent complexes (4 total technical replicates).
Randomization	Data randomization is not relevant to this study.
Blinding	No blinding was used for data collection or analysis.

Reporting for specific materials, systems and methods

We require information from authors about some types of materials, experimental systems and methods used in many studies. Here, indicate whether each material, system or method listed is relevant to your study. If you are not sure if a list item applies to your research, read the appropriate section before selecting a response.

Materials & experimental systems

n/a	Involved in the study	
☒	☐	Antibodies
☐	☒	Eukaryotic cell lines
☒	☐	Palaeontology
☒	☐	Animals and other organisms
☒	☐	Human research participants
☒	☐	Clinical data

Methods

n/a	Involved in the study	
☒	☐	ChIP-seq
☒	☐	Flow cytometry
☒	☐	MRI-based neuroimaging

Eukaryotic cell lines

Policy information about cell lines

Cell line source(s)	CHO-S, HEK293F
Authentication	commercial cell lines
Mycoplasma contamination	Mycoplasma testing is not performed, nor relevant as we are purifying protein from the conditioned media and not performing any cell based assays. The resulting protein is the same regardless of the cells mycoplasma status.
Commonly misidentified lines (See ICLAC register)	*Name any commonly misidentified cell lines used in the study and provide a rationale for their use.*
