# Supplementary material for: Structural analysis of lecithin:cholesterol acyltransferase bound to high density lipoprotein particles
Source: Commun Biol. 2020 Jan 15;3:28. doi: 10.1038/s42003-019-0749-z (PMC6962161; doi:10.1038/s42003-019-0749-z)
Supplement: Supplementary file 3 — Peer Review File [file 42003_2019_749_MOESM3_ESM.pdf]

This manuscript has been previously reviewed at another Nature Research journal. This document only contains reviewer comments and rebuttal letters for versions considered at Communications Biology.

REVIEWERS' COMMENTS:

Reviewer #1 (Remarks to the Author):

Adequately addressed all my original comments. Recommend acceptance.
